# Global molecular evolution and phylogeographic analysis of barley yellow dwarf virus based on the cp and mp genes

**DOI:** 10.1186/s12985-023-02084-1

**Published:** 2023-06-20

**Authors:** Shiqing Wei, Guoliang Chen, Hui Yang, Liang Huang, Guoshu Gong, PeiGao Luo, Min Zhang

**Affiliations:** 1 College of Agronomy, Sichuan Agricultural University, Chengdu, 611130 China; 2 State Key Laboratory for the Biology of Plant Diseases and Insect Pests, Institute of Plant Protection, Chinese Academy of Agricultural Sciences, Beijing, 100193 China

**Keywords:** Barley yellow dwarf virus, Phylogenetic analysis, Viral population history, Phylogeography, Bayesian tip-association significance

## Abstract

Barley yellow dwarf virus (BYDV) has caused considerable losses in the global production of grain crops such as wheat, barley and maize. We investigated the phylodynamics of the virus by analysing 379 and 485 nucleotide sequences of the genes encoding the coat protein and movement protein, respectively. The maximum clade credibility tree indicated that BYDV-GAV and BYDV-MAV, BYDV-PAV and BYDV-PAS share the same evolutionary lineage, respectively. The diversification of BYDV arises from its adaptability to vector insects and geography. Bayesian phylogenetic analyses showed that the mean substitution rates of the coat and movement proteins of BYDV ranged from 8.327 × 10^− 4^ (4.700 × 10^− 4^–1.228 × 10^− 3^) and 8.671 × 10^− 4^ (6.143 × 10^− 4^–1.130 × 10^− 3^) substitutions/site/year, respectively. The time since the most recent common BYDV ancestor was 1434 (1040–1766) CE (Common Era). The Bayesian skyline plot (BSP) showed that the BYDV population experienced dramatic expansions approximately 8 years into the 21st century, followed by a dramatic decline in less than 15 years. Our phylogeographic analysis showed that the BYDV population originating in the United States was subsequently introduced to Europe, South America, Australia and Asia. The migration pathways of BYDV suggest that the global spread of BYDV is associated with human activities.

**Supplementary Information:**

The online version contains supplementary material available at 10.1186/s12985-023-02084-1.

## Background

Barley yellow dwarf virus (BYDV) is a viral disease that has a serious effect on grain crops worldwide; these viral infections may reduce wheat yields by an average of 11–33% and sometimes by up to 80% [1, 2]. In the 1890s, BYDV was widespread in the American Midwest [3]. Initially, the disease was restricted solely to oat crops in the midwestern and eastern United States, but it subsequently became widely distributed in the United States and was discovered to affect wheat, rye, barley, and other cereal species [4–6].

In a seminal paper on the ecological study of Barley yellow dwarf (BYD), Oswald and Houston identified BYDV as a new positive-sense ssRNA virus that is persistent and cyclically transmitted by aphids as the pathogenic agent of BYDV [5, 7]. BYDV is a member of the genus *Luteovirus* in the family Luteoviridae [8, 9]; current data do not show that it can be transmitted mechanically or via seeds [6]. Although at least 25 aphid species have been reported as BYDV carriers [10–12], each virus displays a high degree of vector specificity among different aphid species. According to the International Committee on Taxonomy of Viruses (ICTV), BYDVs are divided into seven different or unassigned genera (BYDV-PAV, BYDV-MAV, BYDV-PAS, BYDV-KerII, BYDV-KerIII, BYDV-SGV and BYDV-GPV) in the family Luteoviridae [13]. Although, five of these species have been classified as strains of BYDV in the *Luteovirus* genus (BYDV-PAV, BYDV-MAV, BYDV-PAS, BYDV-KerII and BYDV-KerIII) on the basis of genetic structure and serological and evolutionary relationships [12, 14, 15], but some reports believe that they are five distinct species, not subspecies of BYDV. *Rhopalosiphum padi* and *Sitobion avenae* efficiently transmit the most common virus, BYDV-PAV, BYDV-MAV, BYDV-SGV, and BYDV-Ker (KerII, KerIII) were found to be transmitted most efficiently by *Si. avenae*, *Schizaphis graminum*, and *R. padi*, respectively [15, 16]. BYDV-GAV can be effectively spread by *Si. avenae* and *Sc. graminum* and is considered a subspecies of the barley yellow dwarf virus MAV [17]. BYDV-GPV is a unique and widespread strain in China that shows no serological relationship with American strains. It is transmitted by *R. padi* and *Sc. graminum* [12].

The genome of BYDV is approximately 5700 nt, and different strains exhibit different genome sizes [12, 18]. The genome harbours six open reading frames (ORFs). ORF2 is solely expressed fused to ORF1 via low-frequency − 1 ribosomal frameshifting in the overlapping region to encode the RNA-dependent RNA polymerase (RdRp) [19]. ORF3 and ORF4 encode the virion assembly protein (coat protein, CP) and cell-to-cell movement protein (movement protein, MP), respectively. ORF5 is fused to CP in a readthrough domain (RTD), which is necessary for transmission via aphids. The functionality of ORF6 near the 3’ may encode viral suppressors of RNA silencing [9, 20].

As researchers have increasingly studied BYDV, we have obtained a deeper understanding of the evolutionary pattern and genetic characteristics of this virus. BYDV-PAV is the most influential genus of BYDV, and the descriptions of several species or subspecies within BYDV-PAV, including BYDV-PAV-I, BYDV-PAV-II (formerly BYDV-PAS) and PAV-IIIa/IIIb, differ as a result of widespread recombination events [21]. More importantly, the results of Bayesian evolutionary analysis show that the mutation of BYDV-PAV may arise from geographic, vector insect and host adaptation and that the evolutionary rate of BYDV-PAV under the action of purifying selection is similar to that of other RNA viruses [22, 23]. These reports have provided us with a deeper understanding of the virus, and the complicated evolutionary mechanism of BYDV has important implications for controlling the effects of the virus in agricultural production.

Unexpectedly, we did not identify the BYDV-GAV strain according to the BYDV classification standard of the ICTV. In addition, one study has surprisingly shown that the BYDV population responsible for the epidemic on the Kerguelen Islands, in the absence of carrier aphids, includes BYDV-KerII and BYDV-KerIII strains [6]. In fact, an inherent characteristic of the virus transmitted by aphids is that it has difficulties effectively spreading across geographic barriers. Nevertheless, an increasing number of reports have confirmed that the dispersal patterns of viruses may be associated with multiple human-mediated factors [14, 24, 25].

RNA viruses exhibit a high mutation rate, rapid replication dynamics, and large virus populations; at the same time, due to the influence of genetic drift, gene flow and natural selection, the evolutionary characteristics and population genetic structure of viruses tend to become more complicated [26]. Moreover, BYDV is restricted by geographical barriers. It is necessary to study the geographic range, epidemiological routes and possible evolutionary mechanisms of BYDV. However, knowledge of the evolutionary biology of BYDV, particularly at a transnational scale, is relatively limited compared to that of other important plant viruses, such as potato virus Y (PVY) and turnip mosaic potyvirus (TuMV) [24, 27, 28]. Therefore, we wanted to give more attention to the evolutionary and genetic characteristics of the BYDV strains and their population histories beyond those of BYDV-PAV alone. Additionally, we put forward some suggestions regarding the classification status of BYDV-GAV.

## Materials and methods

### Sampling and Sequencing

Samples of leaves from wheat (*Triticum aestivum*) were randomly collected across the main wheat-producing regions in Sichuan Province, China in 2021. Two polyclonal antibodies raised against BYDV-PAV and BYDV-MAV and a double antibody sandwich enzyme-linked immunosorbent assay (DAS-ELISA) were used for viral detection in the collected wheat leaves [29]. Twenty-three wheat leaf samples that reacted positively with the BYDV-PAV polyclonal antibody were stored at -80 °C for later use, and some of the BYDV-PAV-infected leaves were used as feeding materials for BYDV transmission vector aphids. After 5 days, the aphids that fed on the infected leaves were placed on healthy wheat leaves until the leaves showed signs of yellowing.

Total RNA was extracted from leaf tissue using TRIzol reagent and was reverse transcribed according to the manufacturer’s instructions (Invitrogen, Carlsbad, CA, United States). The cp gene was amplified using two primers designed from highly conserved regions of BYDV-PAV genomes (AY855920). PCR amplifications were conducted in a total volume of 50 µL containing 2 µL of template cDNA, 10 µL 5×PrimeSTAR Buffer (Mg^2+^ Plus), 1 µL of dNTP Mixture (10 mM each), 2 µL of forward primer (5´-GGATATGGAACAGATGAGCGCCTT-3´), 2 µL of reverse primer (5´-GGATCGGAGTAATATCAACTCGGGA-3´), 0.5 µL of PrimeSTAR HS DNA Polymerase (2.5 U/µl), and 32.5 µL of double-distilled water (ddH_2_O). The PCR program conditions were as follows: After an initial denaturation step at 94 °C for 3 min, 35 cycles were performed consisting of three steps: denaturation at 94 °C for 30 s, annealing at 50 °C for 30 s, and extension at 72 °C for 50 s. The final elongation step was performed at 72 °C for 5 min. PCR products were electrophoresed on 1.0% agarose gels in Tris-acetate-EDTA (TAE) buffer and visualized under UV illumination after staining with ethidium bromide (0.5 mg/mL). PCR products were purified using a QIAAquick Gel Extraction kit (TianGen, Beijing), ligated into the pGADT7-T vector (LMAI Bio, Shanghai), and transformed into *Escherichia coli* strain DH5a cells. The recombinant plasmids were purified, and at least three cDNA clones were sequenced to ensure consensus in both directions; this was carried out by Sangon Biotech Co., Ltd. (Shanghai, China).

### Dataset

By October 2021, we had searched and obtained all the BYDV cp and mp genes complete genome sequences from the GenBank database of the National Center for Biotechnology Information, and these two datasets including BYDV-GPV and BYDV-SGV isolates. Together with our 7 newly obtained isolates of the cp gene complete genome sequences, the multiple alignment of nucleotide sequences was conducted with MAFFTv7 software [30]. Testing for potential recombinant sequences was conducted using seven software programs: RDP, GENECONV, BOOTSCAN, Maximum Chi-Square (MAXCHI), CHIMAERA, 3SEQ and Sister Scanning (SISCAN), implemented with the RDP 4.95 suite [31]. The standard Bonferroni correction was used to set the maximum acceptable p cut-off value to 0.01, with the remaining settings left as the defaults. Recombinants were removed from the subsequent analysis, and we ultimately obtained 426 sequences of the cp gene (including 42 BYDV-GPV and 5 BYDV-SGV isolates) and 534 sequences of the mp gene (including 42 BYDV-GPV and 5 BYDV-SGV isolates) for phylogenetic analysis (Supplementary Table 1).

We reconstructed a phylogenetic tree for BYDV (including BYDV-GPV and BYDV-SGV) using maximum likelihood and Bayesian analysis under the best-fit substitution model (both CP and MP are HKY + *G*), which was selected using the Bayesian information criterion with the PartitionFinder software [32] implemented in PhyloSuite [33–35]. The results indicated that BYDV-GPV was distinct from other BYDV isolates (i.e., BYDV-PAV, BYDV-PAS, BYDV-MAV, BYDV-GAV, BYDV-SGV) and was separated by Soybean dwarf virus (SbDV, NC_003056), Potato leafroll virus (PLRV) and others outgroup (Supplementary Fig. 1, Supplementary Fig. 2). There were three different evolutionary lineages for all isolates other than BYDV-GPV and the outgroups, the isolates of BYDV-SGV alone form the first lineage, the isolates of BYDV-MAV and BYDV-GAV clustered on one branch to form second lineage, and isolates of BYDV-PAV and BYDV-PAS also aggregated on the same branch to form thrid lineage (Supplementary Fig. 1, Supplementary Fig. 2). According to phylogenetic analysis (Supplementary Fig. 1, Supplementary Fig. 2) and reported results [6, 12, 15, 16], BYDV-GPV is not a subspecies of BYDV, and BYDV-PAV, BYDV-PAS, BYDV-MAV and BYDV-GAV are four strains belong to BYDV. So the subsequent analysis of BYDV will not include BYDV-GPV. Although BYDV-SGV may be a subspecies of BYDV, none of these isolates had specific collection dates. Therefore, all subsequent analysis of BYDV included four subspecies: BYDV-PAV, BYDV-PAS, BYDV-MAV and BYDV-GAV. And based on the results of recombination analysis and phylogenetic analysis, the cp and mp gene used for the BYDV analysis consisted of 379 and 485 isolates, respectively. On the basis of 379 isolates of the cp gene and 485 isolates of the mp gene, to ensure more stable results, we selected 10 or more isolates from each geographic region, such that the dataset for analysing the evolutionary relationship between BYDV and geographical location contained 345 cp gene isolates and 458 mp gene isolates. Similarly, less than 10 isolates from the same species were excluded, and the dataset used to analyse the evolutionary relationship between the virus and host species contained 333 cp gene isolates and 415 mp gene isolates.

### Tests for temporal signals

We inferred the evolutionary timescale and substitution rate using a molecular clock calibrated by the sampling times of the sequences in which the temporal signal was evaluated by randomizing the sampling dates over clusters of tips and not over individual tips [36]. The mean substitution rate estimated from the real sampling dates did not overlap with the 95% credibility intervals of rate estimates from 10 replicate datasets with cluster-permuted sampling dates. Moreover, to assess the temporal structure in the sequence data and the substitution rate of the BYDV cp and mp gene, we regressed phylogenetic root-to-tip distances against the date of sampling using TreeTime software, and each analysis was repeated three times to ensure the stability of the results [37]. Each regression yielded a low *r*^2^ value, indicating the presence of rate heterogeneity among lineages. Our results confirmed the presence of a temporal structure in the sequence datasets, allowing us to proceed with our Bayesian molecular dating analyses.

### Temporal dynamics of BYDV

The sequences were analysed using the GTR + *G*_*4*_ substitution model (both CP and MP, excluding BYDV-GPV, BYDV-SGV and the outgroups) substitution model, which was selected using PartitionFinder implemented in PhyloSuite based on the Bayesian information criterion (BIC). We were mindful of the fact that misspecification of the tree prior could result in an incorrect substitution rate, especially during epidemic outbreaks [38]. Therefore, we used marginal likelihood estimates based on path sampling [39] to find the best-fit clock model (including strict and relaxed clocks) and the best-fit tree prior (among the constant size, exponential growth, and Bayesian skyline coalescent) for the dataset with BEAST 1.10 software [40]. An uncorrelated lognormal relaxed clock and Bayesian skyline coalescent tree prior provided the best fit for our datasets in the subsequent analysis (Supplementary Table 2). Four independent Markov chain Monte Carlo (MCMC) analyses were run for 5 × 10^8^ generations, states were sampled every 25,000 steps, and the first 10% of samples were discarded as burn-in. Sufficient sampling was verified by estimating the effective sample sizes (ESS) of all parameters and by inspecting traces with Tracer 1.7 software [41]. The sample sizes have an impact on the diversity of genetic variation [42]. Furthermore, we performed three random samplings on the CP (n = 379) and MP (n = 485) datasets to calculate the most recent common ancestor (MRCA) of BYDV and the evolutionary rates of the cp and mp genes (Supplementary Table 3).

In addition to Bayesian rate estimation, we employed an approximate maximum likelihood approach implemented in TreeTime [37] to infer the evolutionary rate of the cp via the regression of phylogenetic root-to-tip distances against sampling dates.

### Discrete phylogeographic analyses

An asymmetric substitution model with Bayesian random search variable selection options was implemented in BEAST based on the obtained optimal substitution rates for CP and MP (Supplementary Table 3), and significant diffusion rates were tested by means of Bayes factor (BF) calculation, providing inferences of the asymmetric diffusion rates between any two positioning states in 12 regions. The resulting log file was used to calculate the spread of BF between discrete locations and to extract the actual nonzero rate and average metrics for all statistical support routes. Significant migration pathways were identified based on the combination of a BF value greater than 3 and a mean indicator value greater than 0.5. The degree of rate support was as follows: BF > 1,000 indicates decisive support, 100 ≤ BF < 1,000 indicates very strong support, 10 ≤ BF < 100 indicates strong support, and 3 ≤ BF < 10 indicates support [43].

### Phylogeny-Geography Association and Population Structure Analyses

A method accounting for phylogenetic uncertainty in investigating phylogeny-trait correlations with 1000 random permutations of tip locations was implemented with BaTS 2.0 software to estimate the null distribution for each statistic [44]; this was used to calculate values of the association index (*AI*), parsimony score (*PS*) and monophyletic clade (*MC*) size statistics from the posterior sample of trees produced with BEAST1.10 [40] in order to evaluate the associations between the phylogeny and the geographic structure as well as the associations between phylogeny and infected hosts of BYDV. Low *AI* index and *PS* values and high MC scores indicated a strong phylogenetic trait (geography and host) association and low spatial admixture. The average evolutionary rate was obtained according to the best fit to perform molecular clock calibration of the subset to obtain the posterior sample of trees.

Additionally, we used the discriminant analysis of principal components (DAPC) method, which does not rely on the assumptions of Hardy-Weinberg equilibrium and panmixia [45], to investigate the geographic regions and infected hosts of BYDV according to the genetic population structure in R 3.6.3.

## Results

Our datasets for both the cp and mp genes passed the date randomisation test (DRT), which showed that there was no overlap between the true estimate of the evolutionary rate and the 95% confidence interval (CI) generated from 10 random datasets (Supplementary Fig. 3). This indicates that the dataset had a sufficient time signal for reliable Bayesian tip analysis. The linear relationship between sampling time root-to-tip distance of all cp gene datasets and one mp gene datasets (n = 415) also confirmed that the datasets exhibited time signals (Supplementary Fig. 2).

### Evolutionary rates and timescales

**Fig. 1:**
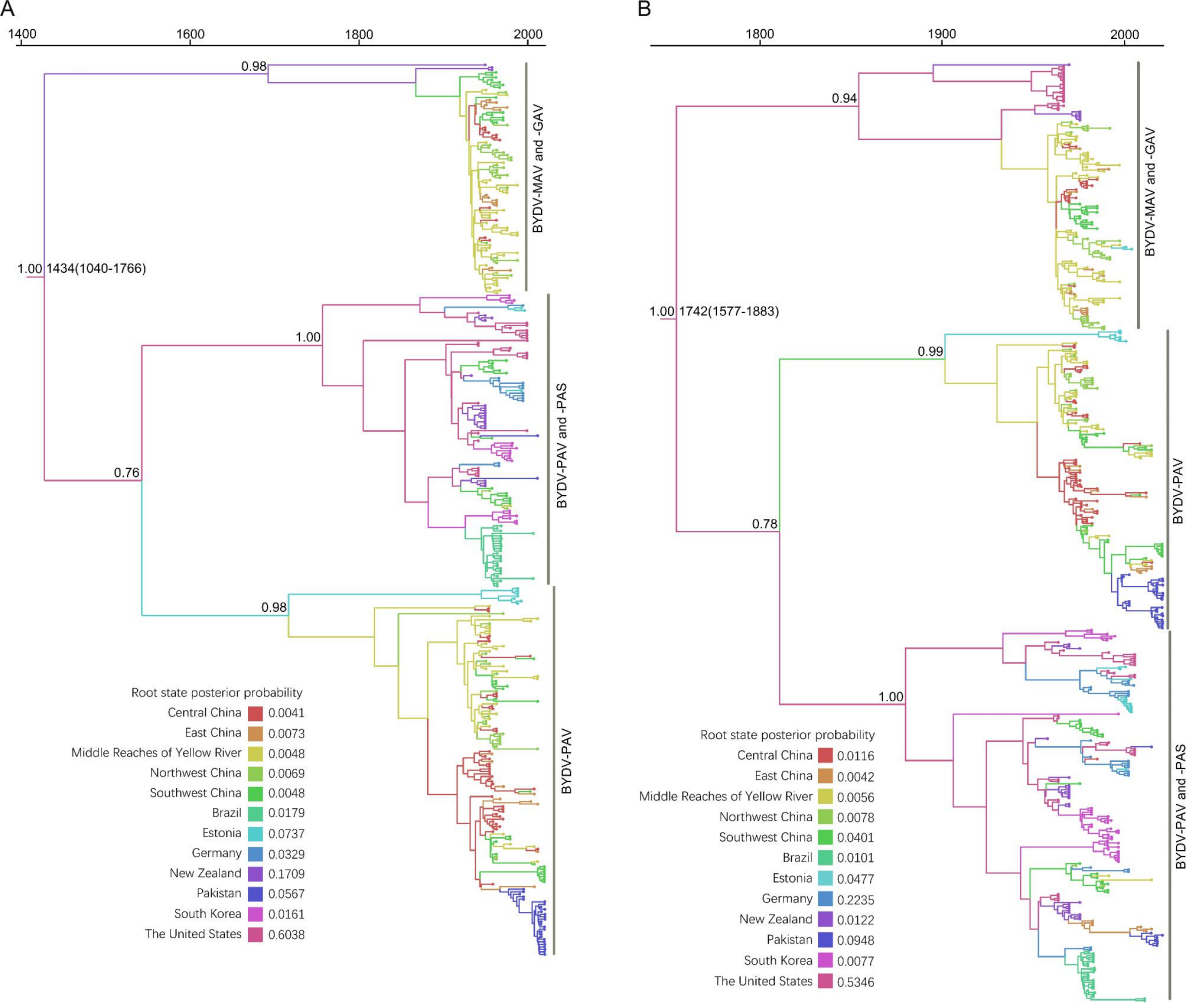
Time-scaled maximum clade credibility tree of barley yellow dwarf virus inferred from the coat protein (**A**) and movement protein (**B**). The tree topologies have been chosen to maximize the product of node posterior probabilities. Branch lengths are scaled according to time, as shown by the horizontal axis. Branch colours denote inferred regions. The root state posterior probabilities of the geographic regions are shown in each inset panel

Bayesian skyline coalescent tree priors and uncorrelated lognormal relaxed clocks provided the best fit for all of the datasets (Supplementary Table 2). The evolutionary rate and MRCA of the BYDV cp and mp genes were calculated using three datasets. There were differences in the results obtained from the different datasets, as well as in the results obtained with Bayesian and with maximum likelihood methods (Supplementary Table 3). When the geographic origin of each isolate was taken into account by the Bayesian method, the calculated genes had a faster evolutionary rate and a later MRCA [46]. In addition, to obtain more accurate evolutionary rates and MRCA determinations for cp and mp genes, three more randomized datasets were generated based on the initial dataset (CP: n = 379, MP: n = 485). Among the six datasets for the mp gene, only one dataset (n = 415) showed a relatively small *r*^2^ value in root-to-tip distance statistics, and the dataset also passed the DRT, with the result that the evolutionary rate of the mp gene was 8.671 × 10^− 4^ substitutions/site/year (95% credibility interval: 6.143 × 10^− 4^–1.130 × 10^− 3^), and the MRCA was 1742 CE (95% credibility interval: 1577 CE–1883 CE, Supplementary Table 3). In contrast the *r*^2^ values of each dataset for the cp were relatively small, so we selected the dataset with the most isolates and calculated that the evolutionary rate of the cp was 8.327 × 10^− 4^ subs/site/year (95% credibility interval, 4.700 × 10^− 4^–1.228 × 10^− 3^). Although the evolution rates of cp gene and mp genes are similar, the MRCA obtained from CP dataset was 1434 CE (95% credibility interval: 1040 CE–1766 CE), which is obviously earlier than that from the MP dataset (Fig. 1, Supplementary Table 3).

We used the obtained optimal evolutionary rate to calibrate the molecular clock for datasets with discrete geographical characteristics. The maximum-clade credibility trees inferred from the CP and MP datasets shared very similar topologies, and all isolates could be separated into two evolutionary lineages: BYDV-PAV and BYDV-MAV (Fig. 1). BYDV-MAV and BYDV-GAV isolates were clustered in the same clade, and all BYDV-PAV and BYDV-PAS isolates were clustered together. The lineage of BYDV-PAV was further divided into two branches, one of which contained BYDV-PAV and BYDV-PAS strains, with isolates mainly from the Americas and Europe, and another branch contained only BYDV-PAV, with isolates mainly from Asia (Fig. 1). Our Bayesian analysis places the root of the trees in the United States with a higher posterior probability than in other regions, and similarly, the other 10 datasets MP and CP yielded the same results (Fig. 1, Supplementary Table 4). Although the datasets of the mp gene have more isolates from different regions (Supplementary Table 4).

### Global migration pattern of BYDV

**Fig. 2:**
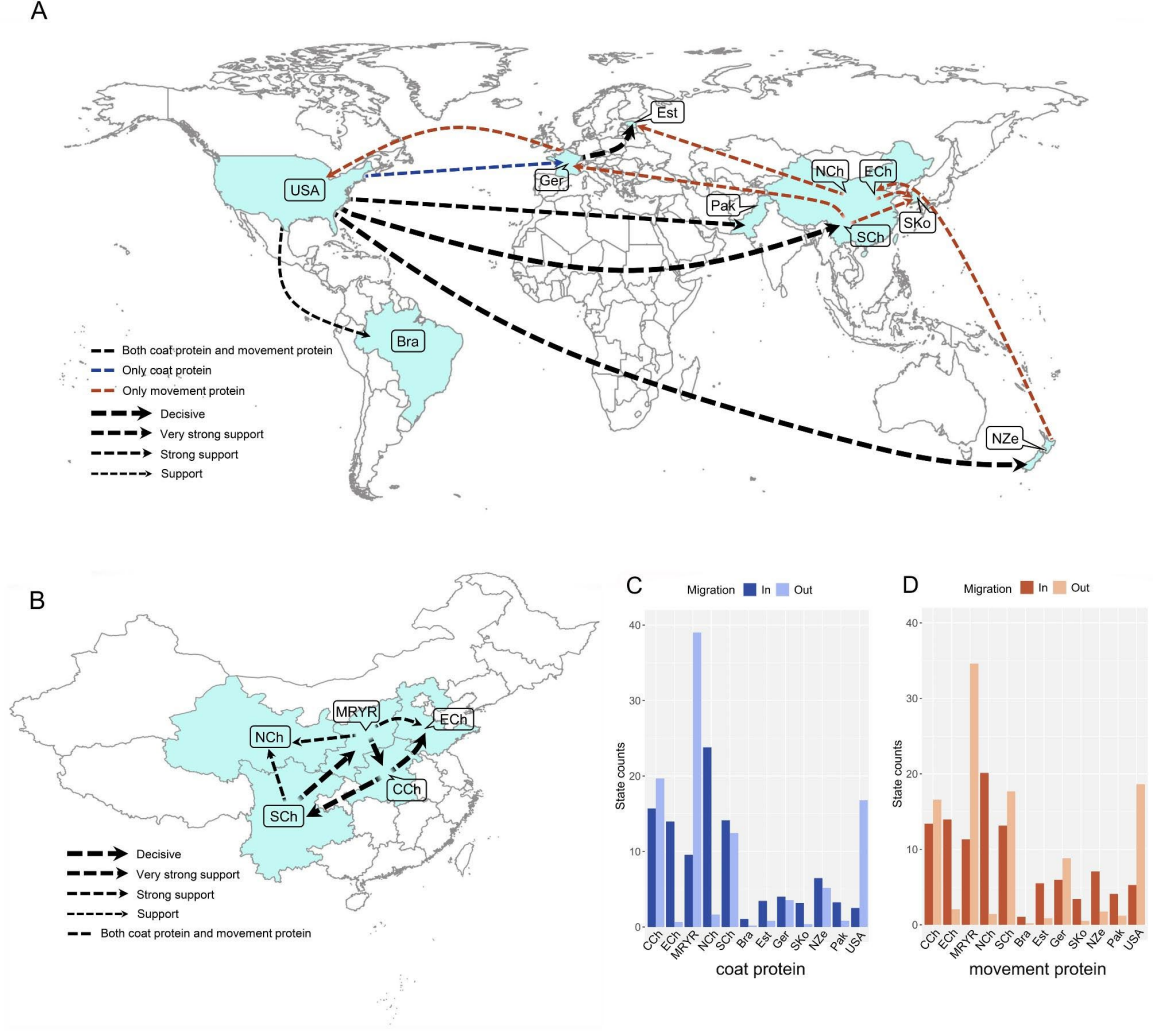
Phylogeographic reconstruction of the spread of barley yellow dwarf virus. (**A**) Supported global spatial diffusion pathways and (**B**) internal pathways within China and (**C**) histogram of the total number of location-state transitions inferred from CP and MP. Bra, Brazil; CCh, Central China; ECh, East China; Est, Estonia; Ger, Germany; MRYR, Middle Reaches of Yellow River; NCh, Northwest China; NZe, New Zealand; Pak, Pakistan; SCh, Southwest China; Sko, South Korea; USA, The United States

Bayesian phylogeographic analysis using the cp and mp genes supported 13 and 18 migration pathways of BYDV in spatial diffusion, respectively (Fig. 2A, Supplementary Table 5). There were 12 migration pathways between different countries, among which 4 pathways originating in the United States were supported by both the CP and MP datasets, spreading to Brazil, New Zealand, Pakistan and Southwest China (Guizhou, Sichuan and Yunnan provinces). There is also a migration pathway supported by the CP dataset originating in the United States and spreading to Germany, while the migration pathway from Germany to the United States is supported by the MP dataset. In addition to the United States as an origin point, there were another 5 pathways originating in other regions supported by the MP dataset. One pathway from New Zealand and spreading to Eastern China (Hebei and Shandong provinces), four pathways originating in different regions of China, two pathways from Southwest China spread to Germany and South Korea, one pathway from East China to South Korea, and the other pathway from Northwest China (Gansu, Ningxia and Qinghai provinces) to Estonia. There was also a migration pathway spreading to Estonia supported by CP and MP datasets, with the emigration region being Germany (Fig. 2A, Supplementary Table 5).

Our statistical results showed seven migration pathways supported by CP and MP datasets that spread within China, and three pathways that spread from the middle reaches of the Yellow River (Shaanxi and Shanxi provinces) to Central China, Eastern China and Northwest China. Two pathways from Southwest China spread to Central China and Northwest China and two pathways from Central China spread to Eastern China and Southwest China (Fig. 2B, Supplementary Table 5).

The inferred spatial dynamics of BYDV suggest that the United States and China acted as important sources for epidemics that emerged in other countries, and this was also supported by the state change counts (that is, the number of geographical state transition/year). Migration from the United States and China being was greater than from any other geographic region included in our analysis, and the migration from the United States is much larger than to the United States (Fig. 2C, D). However, there was much less emigration from than immigration to Brazil, Pakistan, Estonia, South Korea and New Zealand (Fig. 2C, D). In China, the middle reaches of the Yellow River, Central China and Southwest China were the main emigration regions, while East China and Northwest China were the main immigration regions (Fig. 2C, D).

### Demographic history of BYDV

**Fig. 3:**
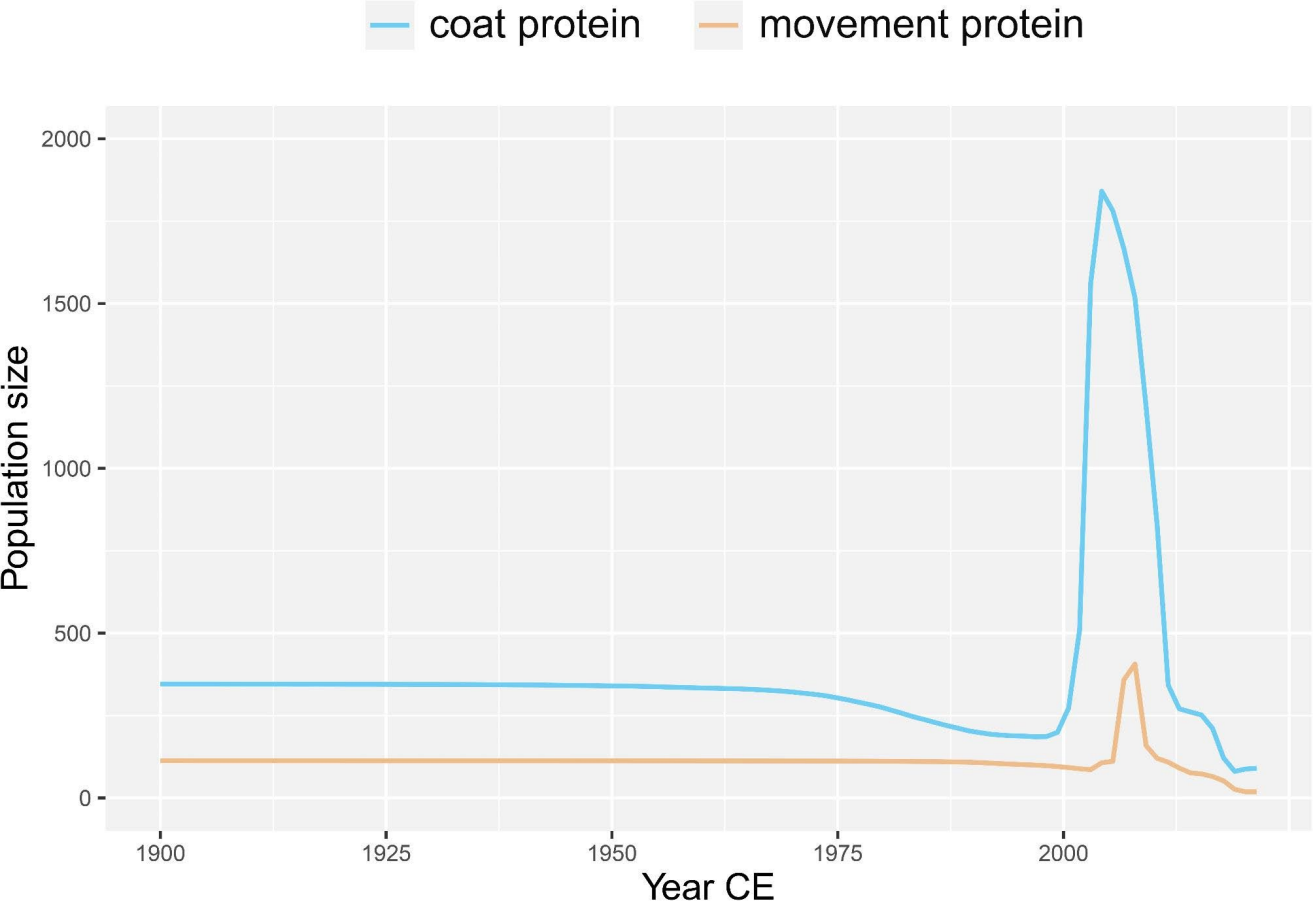
Bayesian skyline plots of barley yellow dwarf virus inferred from CP and MP, showing population size (*y*–axis) through time (*x*–axis)

A coalescence-based BSP using the cp and mp genes revealed an explicit demographic history of the BYDV populations (Fig. 3), showing that the BYDV population remained small and stable before approximately the mid-1970s. After entering the 21st century, the BYDV population experienced a dramatic expansion in approximately 5 years, followed by a dramatic contraction for more than 10 years, and at present, the population of the virus may remain small and stable. In addition, the results based on the cp gene suggest that there may be a slight decline in the BYDV population over a period of approximately 25 years after the mid-1970s.

### Geographic structure patterns of BYDV

The strain structure of BYDV was apparent in the maximum clade credibility (MCC) trees and phylogenetic trees (Fig. 1, Supplementary Fig. 1), but the host and geographical structure were not evident. Therefore, we investigated the global trait association of phylogeography and host structure with the evolution of cp and mp genes (*AI*, p < 0.001 and *PS*, p < 0.001, Table 1). Significant population subdivision was observed for the eight geographic regions according to the *MC* statistic for cp and mp genes. We observed four significant population subdivisions among the four hosts (*Avena sativa*, *Hordeum vulgare*, *Microlaena stipoides* and *Triticum aestivum*) of the cp gene, and only two significant population subdivisions of the mp gene in *A. sativa* and *T. aestivum* among the four hosts (Table 1).

**Table 1:** phylogeny–trait association analysis for the phylogeographic structure of barley yellow dwarf virus using Bayesian tip-association significance testing

Genes	Traits	Statistic	Isolates	Observed mean (95% HPD CIs)	Null mean (95% HPD CIs)	*P*-value
Coat protein	Regions	AI		9.594 (8.060-11.171)	23.771(22.505–24.884)	< 0.001
		PS		69.683 (65.000–74.000)	133.948(131.112-136.113)	< 0.001
		MC (Brazil)	25	4.892(4.000–6.000)	1.450(1.032–2.001)	0.010
		MC (China)	218	11.213(10.000–15.000)	6.992(5.191–9.688)	0.040
		MC (Estonia)	10	2.105(2.000–3.000)	1.101(1.000-1.557)	0.040
		MC (Germany)	12	4.655(4.000–6.000)	1.174(1.000-1.971)	0.010
		MC (New Zealand)	20	7.198(7.000–8.000)	1.265(1.004–1.992)	0.010
		MC (Pakistan)	18	2.290(2.000–3.000)	1.215(1.001–1.981)	0.030
		MC (South Korea)	29	3.256(2.000–5.000)	1.554(1.060–2.035)	0.020
		MC (The United States)	24	7.000(7.000–7.000)	1.484(1.043–2.003)	0.010
	Host	AI		4.339(3.448–5.242)	13.925(12.990-14.925)	< 0.001
		PS		31.064(29.000–34.000)	70.339(68.528–71.874)	< 0.001
		MC (*Avena sativa*)	39	5.957(6.000–6.000)	1.874(1.394–2.284)	0.010
		MC (*Hordeum vulgare*)	24	10.409(9.000–12.000)	1.466(1.082–2.007)	0.010
		MC (*Microlaena stipoides*)	10	7.000(7.000–7.000)	1.070(1.000-1.237)	0.010
		MC (*Triticum aestivum*)	260	63.165(36.000–67.000)	11.634(9.076–15.605)	0.010
Movement protein	Regions	AI		11.031(9.296–12.790)	32.756(31.348_33.952)	< 0.001
		PS		101.499(96.000-107.000)	207.387(204.936-209.816)	< 0.001
		MC (Brazil)	25	2.273(1.000–4.000)	1.283(1.027–1.997)	0.030
		MC(China)	242	18.866(18.000–19.000)	5.400(4.419–6.684)	0.010
		MC (Estonia)	20	2.441(2.000–4.000)	1.265(1.016–1.994)	0.040
		MC (Germany)	22	3.463(2.000–6.000)	1.290(1.016–1.987)	0.010
		MC (New Zealand)	29	2.757(2.000–3.000)	1.405(1.081-2.000)	0.010
		MC (Pakistan)	34	6.100(6.000–7.000)	1.564(1.185–2.070)	0.010
		MC (South Korea)	35	5.798(4.000–7.000)	1.589(1.178–2.071)	0.010
		MC (The United States)	51	8.082(7.000–9.000)	1.973(1.587–2.526)	0.010
	Host	AI		13.562(11.853–15.271)	17.656(16.466–18.641	< 0.001
		PS		83.743(80.000–87.000	97.639(95.001–99.535	< 0.001
		MC (*Avena sativa*)	61	7.122(7.000–8.000)	2.112(1.832–2.583)	0.010
		MC (*Hordeum vulgare*)	30	2.044(2.000–3.000)	1.487(1.177–2.015)	0.090
		MC (*Microlaena stipoides*)	11	1.576(1.000–3.000)	1.081(1.000-1.270)	1.000
		MC (*Triticum aestivum*)	313	21.495(21.000–25.000)	10.996(8.608–4.087)	0.020

The DAPC scatter plots indicated that the Brazilian host population was relatively distinct from the other populations along the first discriminant function axis (Supplementary Fig. 4A, Supplementary Fig. 5A), while the *M. stipoides* host populations were relatively distinct from the other populations along the first discriminant function axes (Supplementary Fig. 4B, Supplementary Fig. 5B). On the second discriminant axis, the populations from Pakistan were distant from the populations in other regions, and the populations from China and South Korea were also distant from the other regions. The populations of *A. sativa*, *H. vulgare* and *T. aestivum* were different on the second discriminant axis, but they were not very clearly separated, while the populations from the hosts *H. vulgare* and *T. aestivum* of the MP gene clustered together on the second discriminant axis (Supplementary Fig. 4B, Supplementary Fig. 5B).

## Discussion

We used the data obtained after screening all complete BYDV CP and MP sequences retrieved from GenBank and our new sequences to conduct a large-scale system dynamics analysis of the global population of BYDV. At present, BYDV-GAV, BYDV-GPV and BYDV-SGV are not classified under BYDV according to searches for the virus in the ICTV database, and there are differing opinions regarding the division of different BYDV strains [12, 16, 47, 48]. We also obtained results regarding the ownership of BYDV-GAV and BYDV-GPV. For both the cp and mp genes, the phylogenetic trees reconstructed by Bayesian and maximum likelihood methods showed that BYDV-GPV was located on one branch, completely separated from BYDV-PAV, BYDV-MAV, BYDV-PAS, BYDV-GAV and BYDV-SGV (Supplementary Fig. 1). In contrast, BYDV-PAV, BYDV-PAS, BYDV-MAV and BYDV-GAV were clustered into one branch other than BYDV-SGV, BYDV-GPV and the outgroups (Supplementary Fig. 1), and were further clustered into two different branches (Fig. 1, Supplementary Fig. 1). This seems to suggest that BYDV-PAV, BYDV-PAS, BYDV-MAV, and BYDV-GAV should be one species. A previous report showed that the lowest identity of the BYDV-PAV cp gene with BYDV-MAV was 73%, and the highest identity with BYDV-GPV was 61% [49]. Another report suggested that the four strains of BYDV-PAV, -PAS, GAV, and -MAV had close identity, while they had distant identity to BYDV-RMV [14]. Furthermore, some reports have shown that BYDV-PAV, BYDV-PAS, BYDV-MAV, and BYDV-GAV congregate in the same evolutionary branch outside the outgroups [14, 16], which is consistent with our results. Based on our results and previous reports [12, 14–18, 49], our suggestion is that BYDV-GAV, BYDV-PAV, BYDV-PAS and BYDV-MAV should be grouped into four strains of BYDV.

Genetic drift, gene flow and natural selection affect the mutation of viruses and determine the genetic structure of species populations [50]. Genetic recombination is also an important evolutionary phenomenon of plant viruses [51]. Previous studies have shown that recombination and natural selection are important driving forces for the evolution and differentiation of BYDV [22, 52, 53]. Our maximum-clade credibility trees show that BYDV initially diverged into two different evolutionary lineages (Fig. 1), and this divergence seems to be caused by different aphids that spread the virus. BYDV infects aphids in a cyclical and sustainable manner, and geminivirus infects aphids, whiteflies and other vectors in a cyclical and sustainable manner. The geminivirus coat protein protects the genome in the vector´s alimentary and circulatory systems [54]. Existing reports suggest that the CP and CP-RTP proteins confer highly specific aphid transmission properties [55]. Therefore, we deduce that in the process of BYDV evolution, to better expand the population, the virus had an inseparable relationship with its vector insects, and thus evolved two different strains: BYDV-PAV and -MAV. Biological and abiotic factors associated with pathogens can individually and interactively affect the extent of genetic drift, gene flow, and selection, thereby influencing the generation and maintenance of spatial population structure [56]. Directional selection of pathogen biology, physical environments, and the ways of human intervention methods during and after agricultural production can drive the rapid accumulation of adaptive genetic differentiation in plant pathogen populations [57].

The genetic variability of many viruses is related to the geographical origin of the viral isolates [27, 58, 59]. As expected, in our study the evolution of BYDV was found to be strongly related to geography (Table 1, Supplementary Fig. 4, Supplementary Fig. 5). Since different regions have different external environments, including varying altitudes and meteorological conditions, this relationship may be a result of evolutionary adaptation of the virus being driven by geographic location. This environmental factor may have led to the specific evolution of the BYDV-MAV strain into the BYDV-GAV strain in China and led it to remain there for a long time. Furthermore, the transmission of this virus by aphids is restricted by the geographical barriers that exist between different regions. In China, the dominant species of wheat aphid mainly include *R. padi*, *Sc. graminum* and *Si. Avenae* (Ministry of Agriculture and Rural Affairs of China), which can effectively transmit BYDV-PAV and -GAV. However, no BYDV-GAV strain has yet been found in Sichuan Province. The dominant species of wheat aphid in Sichuan Province is *R. padi* (Sichuan Academy of Agricultural Sciences), which can effectively transmit BYDV-PAV but not BYDV-GAV. Another important point is that the major wheat producing areas in Sichuan Province are located in the Chengdu Plain, which is surrounded by a natural barrier to aphid migration. Host-driven adaptation could affect the diversification of viral isolates [27, 60]. It has been reported that the diversity of the BYDV-PAV population may be related to geographic adaptations as well as by host-driven adaptation [52]. As expected, our results also suggest that genetic diversity in BYDV is associated with the host, but that different associations are expressed by different genes (Table 1, Supplementary Fig. 4B, Supplementary Fig. 5B). This result may be related to the function of proteins translated by different genes. Coat protein is one of the important components of virions, and it also determines the high specificity of aphid transmission [55]. Movement proteins contribute to disease symptoms and facilitate intra-and intercellular movement [61]. Although ORF4 (mp gene) overlaps in its entirety with ORF3 (cp gene), this difference in the function of the encoded proteins was bound to lead to a differentiated evolutionary outcome in the long-term interaction between the virus and the host plant.

A high mutation rate is one of the characteristics of RNA viruses [62], and the evolutionary rate of plant RNA viruses is generally on the order of 10^− 4^ subs/site/year [23, 27, 63]. Previous reports indicate that the evolutionary rate of the cp of the *Luteoviridae* family is 4.3 × 10^− 4^ subs/site/year, the evolutionary rate of the cp of barley yellow dwarf virus is 1.5 × 10^− 3^ subs/site/year [64], and the average evolutionary rate of the BYDV-PAV genome is 3.158 × 10^− 4^ subs/site/year [52]. The results obtained in our study by using the Bayesian method after molecular clock calibration with recombination-free sequences are quite different from the above results. However, it has been reported that different genes from the same virus may evolve at different rates [27, 46]. Our results show that the evolutionary rates of the cp and mp genes of BYDV are basically similar, at 8.327 × 10^− 4^ subs/site/year (95% credibility interval, 4.700 × 10^− 4^–1.228 × 10^− 3^) and 8.671 × 10^− 4^ (95% credibility interval, 6.143 × 10^− 4^–1.130 × 10^− 3^), respectively. This result is not surprising as ORF4 (mp gene) overlaps in its entirety with ORF3 (cp gene).

One of the difficulties in estimating the dates of MRCAs is that plant RNA viruses include many recombinants, so a larger sequence sample is required to reliably estimate these dates [46]. Different datasets will lead to different MRCA results, and different genes will produce different results. Previous reports have suggested that the MRCA of BYDV-PAV was estimated to be between 268 and 4680 years ago [52], and another report showed that the earliest common ancestor of BYDV was in the range of 13-2009 years ago (with RdRp giving more recent dates and RTD producing earlier dates, [64]). Notably, Wu et al. (2011) did not test the temporal signal of the dataset or filter the best fit before analysing the BYDV MRCA data, which the cause of the discrepancy. In particular, misspecification of the tree prior could result in incorrect substitution rates and inaccurate MRCA data [38]. We performed molecular clock calibration on our dataset in a more rigorous way and molecularly dated different datasets with recombination-free cp and mp genes using Bayesian and maximum likelihood methods (Supplementary Table 3). Our results show that the MRCA of BYDV calculated using the cp and mp genes is 1444 CE (95% credibility interval: 1040–1766 CE) and 1742 CE (95% credibility interval: 1577 CE–1883 CE), respectively.

Our results suggest that there is a high probability that BYDV originated in the United States, and the root posterior probabilities for the USA are much higher than those in other regions, whether using the six datasets of the CP or the MP genes (Fig. 1, Supplementary Table 4). Not surprisingly, BYDV was first discovered and reported in the United States, and samples from infected hosts of BYDV were observed in the United States over 100 years ago [3, 7]. An increasing number of reports have shown that multiple human-associated factors can spread the virus across geographic barriers [14, 25, 28, 46, 65]. We reconstructed the migration pathways of BYDV on a global scale and identified multiple migration pathways of BYDV from the USA and China to other regions, indicating that the USA and China have been important hubs for the global spread of this pathogen (Fig. 2, Supplementary Table 5). BYDV has reportedly spread over long distances through maritime trade between Australia and the United States [65]. A recent report also confirmed our result of a migration pathway from Northwest China to Estonia [17]. On the basis of the inference of the geographical origin of the virus and its global migration path, we are more convinced that BYDV originated in the United States. After spreading from the United States to South America, Asia, Australia and Europe, it further spread from China to South Korea, Estonia and Germany. In this way, barley yellow dwarf virus spread around the world, and the population size expanded dramatically. Under different selective pressures from different management patterns, different environments, different resistant varieties, and different farm chemicals, the evolutionary pattern of the barley yellow dwarf virus was specialized in relation to the region and host.

With ongoing research on the virus, we have obtained a profound understanding of how to prevent the virus from causing crop diseases [6]. We performed a molecular evolutionary analysis of BYDV isolates based on two genes, and our findings have provided new insights into the evolutionary history of BYDV. We suggest that BYDV-PAV, BYDV-PAS, BYDV-MAV and BYDV-GAV should be classified as one species named BYDV. Whether BYDV-KerII, BYDV-KerIII and BYDV-SGV belong to BYDV as subspecies has not yet been concluded. BYDV-KerII and BYDV-KerIII isolates lacked nonrecombinant complete cp and mp gene sequences, and our phylogenetic analysis suggests that BYDV-SGV may belong to a new species distinct from BYDV. The evolution of BYDV is related to geography and to its aphid transmission vectors, and it may also be related to the adaptation of its infected hosts. Through pedigree and geographic analysis, we found that BYDV probably originated in the United States and spread to other regions, and that China was the main export region for BYDV. Surprisingly, the population size of this virus expanded dramatically across the globe less than 8 years into the 21st century, followed by a sharp decline less than 15 years later, which is largely related to in-depth scientific research and is consistent with our field investigations (unpublished). There is no doubt that when a new disease causes large-scale losses in agricultural production, effective chemical pesticides, rational agricultural management measures and resistant varieties are usually used in agricultural practices to control the impact of the pathogen. Although we performed a more comprehensive analysis of the population history and evolutionary characteristics of BYDV using numerous isolates of the cp and mp genes, we may need variant information on ORF1 and ORF2 for further evaluation of BYDV evolutionary characteristics and population history.

## Electronic supplementary material

Below is the link to the electronic supplementary material.

Supplementary Material 1

Supplementary Material 2

Supplementary Material 3

Supplementary Material 4

Supplementary Material 5

Supplementary Material 6

## Data Availability

We guarantee the authenticity and availability of all data and materials in the manuscript and the results/data/figures in this manuscript have not been published elsewhere, nor are they under consideration (from you or one of your Contributing Authors) by another publisher.
