# Supplementary material for: Global molecular evolution and phylogeographic analysis of barley yellow dwarf virus based on the cp and mp genes
Source: Virol J. 2023 Jun 20;20:130. doi: 10.1186/s12985-023-02084-1 (PMC10280855; doi:10.1186/s12985-023-02084-1)
Supplement: Supplementary file 1 — Supplementary Material 1 [file 12985_2023_2084_MOESM1_ESM.docx]

Supplementary Material

Supplementary Fig. 1 Phylogenetic tree of barley yellow dwarf virus reconstructed by Maximum likelihood and Bayesian methods inferred from the coat protein (A) and movement protein (B). The color of the branches represents different strains, and the two outer rings are different regions and different hosts with different colors.

Supplementary Fig. 2 Linear regression analysis of the root-to-tip regression of genetic distance against sampling dates for different genomic segments from CP (A) and MP (B) recombination-free sequences.

Supplementary Fig. 3 Date-randomization test for temporal signal in the sequence data. The *x*-axis shows 10 different randomizations of the dates after clustered permutations in the data sets of CP (A) and MP (B), along with the non-randomized data set. The *y*-axis indicates the estimated substitution rate.

Supplementary Fig. 4 Scatterplots resulting from the discriminant analysis of principal components (DAPC) from CP. Individual isolates from the same geographic regions (A) and host (B) are depicted with uniquely coloured shapes and surrounded by 95% inertia ellipses. The PCA and DA eigenvalue inset panels show the overall variability among individuals and the relative capture of variance for each discriminant function, respectively. The *y*- and *x*-axes indicate the first and second discriminant principal components, respectively.

Supplementary Fig. 5 Scatterplots resulting from the discriminant analysis of principal components (DAPC) from MP. Individual isolates from the same geographic regions (A) and host (B) are depicted with uniquely coloured shapes and surrounded by 95% inertia ellipses. The PCA and DA eigenvalue inset panels show the overall variability among individuals and the relative capture of variance for each discriminant function, respectively. The *y*- and *x*-axes indicate the first and second discriminant principal components, respectively.

Supplementary Table. 1 Isolates of BYDV used in this study.

| GenBank ID | Location | Collection date | Host | Strain | Genes |
| --- | --- | --- | --- | --- | --- |
| MK962883 | Australia | 01-Apr-2019 | *Triticum aestivum* | PAV | CP, MP |
| JX067816 | Brazil | Jul-2007 | *Avena sativa* | PAV | CP, MP |
| JX067817 | Brazil | Jul-2007 | *Avena sativa* | PAV | CP, MP |
| JX067818 | Brazil | Jul-2007 | *Avena sativa* | PAV | CP, MP |
| JX067819 | Brazil | Jul-2007 | *Avena sativa* | PAV | CP, MP |
| JX067820 | Brazil | Jul-2007 | *Avena sativa* | PAV | CP, MP |
| JX067821 | Brazil | Aug-2007 | *Triticum aestivum* | PAV | CP, MP |
| JX067824 | Brazil | Aug-2007 | *Avena sativa* | PAV | CP, MP |
| JX067827 | Brazil | Jul-2008 | *Avena sativa* | PAV | CP, MP |
| JX067829 | Brazil | Jul-2008 | *Avena sativa* | PAV | CP, MP |
| JX067831 | Brazil | Jul-2008 | *Triticum aestivum* | PAV | CP, MP |
| JX067833 | Brazil | Jul-2008 | *Avena sativa* | PAV | CP, MP |
| JX067834 | Brazil | Jul-2008 | *Avena sativa* | PAV | CP, MP |
| JX067835 | Brazil | Sep-2008 | *Avena sativa* | PAV | CP, MP |
| JX067837 | Brazil | Jul-2008 | *Avena sativa* | PAV | CP, MP |
| JX067838 | Brazil | Jul-2008 | *Avena sativa* | PAV | CP, MP |
| JX067839 | Brazil | Jul-2008 | *Avena sativa* | PAV | CP, MP |
| JX067840 | Brazil | Jul-2008 | *Avena sativa* | PAV | CP, MP |
| JX067841 | Brazil | Jul-2008 | *Triticum aestivum* | PAV | CP, MP |
| JX067845 | Brazil | Jul-2008 | *Lolium perenne* | PAV | CP, MP |
| JX067847 | Brazil | Oct-2008 | *Triticum aestivum* | PAV | CP, MP |
| JX067850 | Brazil | Oct-2008 | *Zea mays* | PAV | CP, MP |
| JX067853 | Brazil | Jul-2008 | *Avena sativa* | PAV | CP, MP |
| JX067854 | Brazil | Jul-2008 | *Avena sativa* | PAV | CP, MP |
| MT345894 | Brazil | 2018 | *Triticum aestivum* | PAV | CP, MP |
| MT345895 | Brazil | 2018 | *Avena sativa* | PAV | CP, MP |
| China | | | | | |
| EU332314*^b^* | Hubei | 25-Apr-2005 | unknown | PAV | CP, MP |
| EU332334*^a^* | Hubei | 2006 | *Triticum aestivum* | PAV | CP, MP |
| EU386792*^b^* | Hubei | 25-Apr-2005 | *Avena fatua* | GAV | MP |
| EU386806*^b^* | Hubei | 25-Apr-2005 | *Avena fatua* | GAV | CP, MP |
| KP096644 | Hubei | 13-Mar-2009 | *Triticum aestivum* | PAV | CP, MP |
| KP096645 | Hubei | 13-Mar-2009 | *Triticum aestivum* | PAV | CP, MP |
| KP096646 | Hubei | 13-Mar-2009 | *Triticum aestivum* | PAV | CP, MP |
| KP096647 | Hubei | 13-Mar-2009 | *Triticum aestivum* | PAV | CP, MP |
| KP341654 | Hubei | 13-Mar-2009 | *Triticum aestivum* | GAV | MP |
| MN648437 | Hubei | 20-Feb-2017 | *Triticum aestivum* | PAV | CP, MP |
| EU332307*^a^* | Henan | 2006 | *Triticum aestivum* | PAV | CP, MP |
| EU332308*^a^* | Henan | 2004 | *Triticum aestivum* | PAV | CP, MP |
| EU332312*^b^* | Henan | 31-Mar-2005 | *Triticum aestivum* | PAV | CP, MP |
| EU332320*^b^* | Henan | 31-Mar-2005 | *Triticum aestivum* | PAV | CP, MP |
| EU332321*^b^* | Henan | 31-Mar-2005 | *Triticum aestivum* | PAV | CP, MP |
| EU332322*^b^* | Henan | 31-Mar-2005 | *Triticum aestivum* | PAV | CP, MP |
| EU332323*^b^* | Henan | 31-Mar-2005 | *Triticum aestivum* | PAV | CP, MP |
| EU332324*^b^* | Henan | 31-Mar-2005 | *Triticum aestivum* | PAV | CP, MP |
| EU332325*^b^* | Henan | 2005 | *Triticum aestivum* | PAV | CP, MP |
| EU332326*^b^* | Henan | 2005 | *Triticum aestivum* | PAV | CP, MP |
| EU332335*^b^* | Henan | 2006 | *Triticum aestivum* | PAV | CP, MP |
| EU332336*^b^* | Henan | 2006 | *Triticum aestivum* | PAV | CP, MP |
| EU386804*^b^* | Henan | 2004 | *Triticum aestivum* | GAV | CP, MP |
| EU332327*^b^* | Henan | 2005 | *Triticum aestivum* | PAV | CP, MP |
| EU386776*^b^* | Henan | 2004 | *Triticum aestivum* | GAV | CP, MP |
| EU386783*^b^* | Henan | 2004 | *Triticum aestivum* | GAV | CP, MP |
| EU386784*^b^* | Henan | 2004 | *Triticum aestivum* | GAV | CP, MP |
| EU386785*^b^* | Henan | 2005 | *Triticum aestivum* | GAV | CP, MP |
| EU386791*^b^* | Henan | 20-Apr-2005 | *Triticum aestivum* | GAV | CP, MP |
| KP096595 | Henan | 20-Mar-2009 | *Triticum aestivum* | PAV | MP |
| KP096602 | Henan | 05-Apr-2007 | *Triticum aestivum* | PAV | CP, MP |
| KP096603 | Henan | 30-Apr-2007 | *Triticum aestivum* | PAV | CP, MP |
| KP096604 | Henan | 30-Apr-2007 | *Triticum aestivum* | PAV | CP, MP |
| KP096605 | Henan | 30-Apr-2007 | *Triticum aestivum* | PAV | CP, MP |
| KP096608 | Henan | 07-Apr-2007 | *Triticum aestivum* | PAV | CP, MP |
| KP096609 | Henan | 07-Apr-2007 | *Triticum aestivum* | PAV | CP, MP |
| KP096610 | Henan | 07-Apr-2007 | *Triticum aestivum* | PAV | CP, MP |
| KP096628 | Henan | 10-Apr-2008 | *Triticum aestivum* | PAV | CP, MP |
| KP096629 | Henan | 10-Apr-2008 | *Triticum aestivum* | PAV | CP, MP |
| KP096686 | Henan | 01-Apr-2005 | *Triticum aestivum* | PAV | CP, MP |
| KP096687 | Henan | 01-Apr-2005 | *Triticum aestivum* | PAV | CP, MP |
| KP096688 | Henan | 01-Apr-2005 | *Triticum aestivum* | PAV | CP, MP |
| KP096689 | Henan | 01-Apr-2006 | *Triticum aestivum* | PAV | CP, MP |
| KP096690 | Henan | 01-Apr-2006 | *Triticum aestivum* | PAV | CP, MP |
| KP096691 | Henan | 01-Apr-2006 | *Triticum aestivum* | PAV | CP, MP |
| KP096692 | Henan | 01-Apr-2006 | *Triticum aestivum* | PAV | CP, MP |
| KP096695 | Henan | 01-Apr-2006 | *Triticum aestivum* | PAV | CP, MP |
| KP341551 | Henan | 05-Apr-2007 | *Triticum aestivum* | GAV | CP, MP |
| KP341553 | Henan | 05-Apr-2007 | *Triticum aestivum* | GAV | CP, MP |
| KP341554 | Henan | 05-Apr-2007 | *Triticum aestivum* | GAV | CP, MP |
| KP341579 | Henan | 10-Apr-2008 | *Triticum aestivum* | GAV | CP, MP |
| MN648438 | Henan | 11-Apr-2018 | *Triticum aestivum* | PAV | CP, MP |
| MN648440 | Henan | 10-May-2019 | *Triticum aestivum* | PAV | CP, MP |
| EU386778*^b^* | Hebei | 24-Apr-2004 | *Triticum aestivum* | GAV | CP, MP |
| EU386807*^b^* | Hebei | 2006 | unknown | GAV | CP, MP |
| EU386812*^b^* | Hebei | 2005 | *Triticum aestivum* | GAV | CP, MP |
| KP341610 | Hebei | 18-May-2010 | *Triticum aestivum* | GAV | CP, MP |
| KP341636 | Hebei | 15-May-2011 | *Triticum aestivum* | GAV | CP, MP |
| KP341548 | Hebei | 23-Apr-2007 | *Triticum aestivum* | GAV | CP, MP |
| KP341549 | Hebei | 23-Apr-2007 | *Triticum aestivum* | GAV | CP, MP |
| KP341550 | Hebei | 23-Apr-2007 | *Triticum aestivum* | GAV | CP, MP |
| MN648430 | Hebei | 28-Apr-2018 | *Triticum aestivum* | PAV | CP, MP |
| MN648431 | Hebei | 24-Apr-2019 | *Triticum aestivum* | PAV | CP, MP |
| EU332330*^a^* | Shandong | 2006 | *Triticum aestivum* | PAV | CP, MP |
| EU332331*^a^* | Shandong | 2006 | *Triticum aestivum* | PAV | CP, MP |
| EU386788*^b^* | Shandong | 20-May-2004 | *Triticum aestivum* | GAV | CP, MP |
| EU386789*^b^* | Shandong | 23-May-2005 | *Triticum aestivum* | GAV | CP, MP |
| KP096633 | Shandong | 05-May-2008 | *Triticum aestivum* | PAV | CP, MP |
| KP096632 | Shandong | 05-May-2008 | *Triticum aestivum* | PAV | MP |
| KP096634 | Shandong | 05-May-2008 | *Triticum aestivum* | PAV | MP |
| KP096635 | Shandong | 05-May-2008 | *Triticum aestivum* | PAV | MP |
| KP096649 | Shandong | 13-May-2009 | *Triticum aestivum* | PAV | CP, MP |
| KP341563 | Shandong | 27-Apr-2007 | *Triticum aestivum* | GAV | CP, MP |
| KP341580 | Shandong | 05-May-2008 | *Triticum aestivum* | GAV | CP, MP |
| MN648434 | Shandong | 17-Apr-2017 | *Triticum aestivum* | PAV | CP, MP |
| MN648435 | Shandong | 28-Apr-2018 | *Triticum aestivum* | PAV | CP, MP |
| MN648436 | Shandong | 23-Apr-2019 | *Triticum aestivum* | PAV | CP, MP |
| MN648441 | Shandong | 28-Apr-2018 | *Triticum aestivum* | PAV | MP |
| MN648442 | Shandong | 23-Apr-2019 | *Triticum aestivum* | PAV | MP |
| MN648444 | Shandong | 17-Apr-2017 | *Triticum aestivum* | PAV | MP |
| EU386777*^b^* | Shanxi | 01-Jun-2004 | *Triticum aestivum* | GAV | CP, MP |
| EU386793*^b^* | Shanxi | 11-Apr-2004 | *Triticum aestivum* | GAV | CP, MP |
| EU386796*^b^* | Shanxi | 03-Jun-2005 | *Avena sativa* | GAV | CP, MP |
| EU386797*^b^* | Shanxi | 03-Jun-2005 | *Avena sativa* | GAV | CP, MP |
| KP341632 | Shanxi | 14-May-2010 | *Triticum aestivum* | GAV | CP, MP |
| KP341595 | Shanxi | 11-Jun-2009 | *Triticum aestivum* | GAV | CP, MP |
| KP341625 | Shanxi | 23-Jun-2010 | *Triticum aestivum* | GAV | CP, MP |
| KP341570 | Shanxi | 09-May-2007 | *Triticum aestivum* | GAV | CP, MP |
| KF523380 | Shanxi | 20-May-2013 | *Triticum aestivum* | GAV | CP, MP |
| KP341564 | Shanxi | 14-Jun-2007 | *Triticum aestivum* | GAV | CP, MP |
| KP341566 | Shanxi | 14-Jun-2007 | *Triticum aestivum* | GAV | CP, MP |
| KP341568 | Shanxi | 14-Jun-2007 | *Triticum aestivum* | GAV | CP, MP |
| KP341569 | Shanxi | 14-Jun-2007 | *Triticum aestivum* | GAV | CP, MP |
| KP341572 | Shanxi | 26-Apr-2007 | *Triticum aestivum* | GAV | CP, MP |
| KP341582 | Shanxi | 08-May-2008 | *Triticum aestivum* | GAV | CP, MP |
| KP341594 | Shanxi | 11-Jun-2009 | *Triticum aestivum* | GAV | CP, MP |
| KP341599 | Shanxi | 08-May-2009 | *Triticum aestivum* | GAV | CP, MP |
| KP341624 | Shanxi | 23-Jun-2010 | *Triticum aestivum* | GAV | CP, MP |
| KP341630 | Shanxi | 19-May-2010 | *Triticum aestivum* | GAV | CP, MP |
| KP341631 | Shanxi | 14-May-2010 | *Triticum aestivum* | GAV | CP, MP |
| KP341651 | Shanxi | 01-Jun-2005 | *Triticum aestivum* | GAV | CP, MP |
| KP341653 | Shanxi | 28-Apr-2009 | *Triticum aestivum* | GAV | MP |
| MN648432 | Shanxi | 19-Apr-2018 | *Triticum aestivum* | PAV | CP, MP |
| MN648433 | Shanxi | 16-Apr-2019 | *Triticum aestivum* | PAV | CP, MP |
| MN648443 | Shanxi | 16-Apr-2019 | *Triticum aestivum* | PAV | MP |
| EU332315*^b^* | Shaanxi | 21-Apr-2005 | *Triticum aestivum* | PAV | CP, MP |
| EU332316*^b^* | Shaanxi | 21-Apr-2005 | *Triticum aestivum* | PAV | CP, MP |
| EU332318*^b^* | Shaanxi | 21-Apr-2005 | *Triticum aestivum* | PAV | CP, MP |
| EU332319*^b^* | Shaanxi | 21-Apr-2005 | *Triticum aestivum* | PAV | CP, MP |
| EU386787*^b^* | Shaanxi | 24-Apr-2004 | *Triticum aestivum* | GAV | CP, MP |
| EU386794*^b^* | Shaanxi | 24-Apr-2004 | *Triticum aestivum* | GAV | CP, MP |
| EU386803*^b^* | Shaanxi | 24-Apr-2004 | *Triticum aestivum* | GAV | CP, MP |
| EU386808*^b^* | Shaanxi | 24-Apr-2004 | *Triticum aestivum* | GAV | CP, MP |
| EU386809*^b^* | Shaanxi | 20-Apr-2005 | *Triticum aestivum* | GAV | CP, MP |
| EU386810*^b^* | Shaanxi | 21-Apr-2005 | *Triticum aestivum* | GAV | CP, MP |
| EU386813*^b^* | Shaanxi | 21-Apr-2005 | *Triticum aestivum* | GAV | CP, MP |
| EU402386*^b^* | Shaanxi | 21-Apr-2005 | *Triticum aestivum* | GAV | MP |
| EU402387*^b^* | Shaanxi | 21-Apr-2005 | *Triticum aestivum* | GAV | MP |
| EU402388*^b^* | Shaanxi | 21-Apr-2005 | *Triticum aestivum* | GAV | MP |
| EU402389*^b^* | Shaanxi | 21-Apr-2005 | *Triticum aestivum* | GAV | MP |
| EU402390*^b^* | Shaanxi | 21-Apr-2005 | *Triticum aestivum* | GAV | MP |
| EU402391*^b^* | Shaanxi | 21-Apr-2005 | *Triticum aestivum* | GAV | MP |
| KF523378 | Shaanxi | 30-Apr-2013 | *Triticum aestivum* | GAV | CP, MP |
| KF523379 | Shaanxi | 30-Apr-2013 | *Triticum aestivum* | GAV | CP, MP |
| KF523381 | Shaanxi | 25-May-2013 | *Triticum aestivum* | GAV | CP, MP |
| KP096593 | Shaanxi | 17-Apr-2009 | *Triticum aestivum* | PAV | CP, MP |
| KP096594 | Shaanxi | 17-Apr-2009 | *Triticum aestivum* | PAV | CP, MP |
| KP096616 | Shaanxi | 15-Apr-2007 | *Triticum aestivum* | PAV | CP, MP |
| KP096617 | Shaanxi | 15-Apr-2007 | *Triticum aestivum* | PAV | CP, MP |
| KP096618 | Shaanxi | 15-Apr-2007 | *Triticum aestivum* | PAV | CP, MP |
| KP096650 | Shaanxi | 28-Apr-2009 | *Triticum aestivum* | PAV | CP, MP |
| KP096656 | Shaanxi | 22-Apr-2010 | *Triticum aestivum* | PAV | CP, MP |
| KP096663 | Shaanxi | 18-May-2011 | *Triticum aestivum* | PAV | CP, MP |
| KP096664 | Shaanxi | 18-May-2011 | *Triticum aestivum* | PAV | CP, MP |
| KP096673 | Shaanxi | 01-Apr-2004 | *Triticum aestivum* | PAV | CP, MP |
| KP096674 | Shaanxi | 01-Apr-2004 | *Triticum aestivum* | PAV | CP, MP |
| KP096680 | Shaanxi | 01-May-2005 | *Triticum aestivum* | PAV | CP, MP |
| KP096682 | Shaanxi | 01-May-2005 | *Triticum aestivum* | PAV | CP, MP |
| KP096681 | Shaanxi | 01-May-2005 | *Triticum aestivum* | PAV | CP, MP |
| KP096683 | Shaanxi | 01-May-2005 | *Triticum aestivum* | PAV | CP, MP |
| KP096684 | Shaanxi | 01-Apr-2005 | *Triticum aestivum* | PAV | CP, MP |
| KP096685 | Shaanxi | 01-Apr-2005 | *Triticum aestivum* | PAV | CP, MP |
| KP341573 | Shaanxi | 15-Apr-2007 | *Triticum aestivum* | GAV | CP, MP |
| KP341633 | Shaanxi | 22-Apr-2010 | *Triticum aestivum* | GAV | CP, MP |
| MN648424 | Shaanxi | 13-Apr-2017 | *Triticum aestivum* | PAV | CP, MP |
| MN648426 | Shaanxi | 14-Apr-2019 | *Triticum aestivum* | PAV | CP, MP |
| MN648428 | Shaanxi | 26-Mar-2019 | *Triticum aestivum* | PAV | CP, MP |
| MN648429 | Shaanxi | 23-Apr-2019 | *Triticum aestivum* | PAV | CP, MP |
| EU332309*^b^* | Gansu | 19-Apr-2005 | *Triticum aestivum* | PAV | CP, MP |
| EU332310*^b^* | Gansu | 19-Apr-2005 | *Triticum aestivum* | PAV | CP, MP |
| EU332311*^b^* | Gansu | 19-Apr-2005 | *Triticum aestivum* | PAV | CP, MP |
| EU332313*^a^* | Gansu | 2005 | *Triticum aestivum* | PAV | CP, MP |
| KF523382 | Gansu | 02-Jun-2013 | *Triticum aestivum* | GAV | CP, MP |
| KP096599 | Gansu | 14-Apr-2007 | *Triticum aestivum* | PAV | CP, MP |
| KP096600 | Gansu | 14-Apr-2007 | *Triticum aestivum* | PAV | CP, MP |
| KP096606 | Gansu | 14-Apr-2007 | *Triticum aestivum* | PAV | CP, MP |
| KP096607 | Gansu | 14-Apr-2007 | *Triticum aestivum* | PAV | CP, MP |
| KP096621 | Gansu | 24-Apr-2008 | *Triticum aestivum* | PAV | CP, MP |
| KP096623 | Gansu | 24-Apr-2008 | *Triticum aestivum* | PAV | CP, MP |
| KP096624 | Gansu | 24-Apr-2008 | *Triticum aestivum* | PAV | CP, MP |
| KP096625 | Gansu | 24-Apr-2008 | *Triticum aestivum* | PAV | CP, MP |
| KP096638 | Gansu | 22-Mar-2009 | *Triticum aestivum* | PAV | CP |
| KP096651 | Gansu | 15-Jun-2010 | *Triticum aestivum* | PAV | CP, MP |
| KP096676 | Gansu | 01-Apr-2004 | *Triticum aestivum* | PAV | CP, MP |
| KP096677 | Gansu | 01-Apr-2004 | *Triticum aestivum* | PAV | CP, MP |
| KP096678 | Gansu | 01-Apr-2004 | *Triticum aestivum* | PAV | CP, MP |
| KP096637 | Gansu | 23-May-2009 | *Triticum aestivum* | PAV | CP, MP |
| KP096675 | Gansu | 01-Apr-2004 | *Triticum aestivum* | PAV | CP, MP |
| KP341578 | Gansu | 24-Apr-2008 | *Triticum aestivum* | GAV | CP, MP |
| KP341590 | Gansu | 23-May-2009 | *Triticum aestivum* | GAV | CP, MP |
| KP341593 | Gansu | 22-May-2009 | *Triticum aestivum* | GAV | CP, MP |
| MN648427 | Gansu | 14-Apr-2019 | *Triticum aestivum* | PAV | CP, MP |
| KP341611 | Qinghai | 2010 | *Triticum aestivum* | GAV | CP, MP |
| KP341612 | Qinghai | 23-Jun-2010 | *Triticum aestivum* | GAV | CP, MP |
| KP341613 | Qinghai | 23-Jun-2010 | *Triticum aestivum* | GAV | CP, MP |
| KP341614 | Qinghai | 24-Jun-2010 | *Triticum aestivum* | GAV | CP, MP |
| KP341616 | Qinghai | 24-Jun-2010 | *Triticum aestivum* | GAV | CP, MP |
| KP341639 | Qinghai | 28-Jul-2011 | *Triticum aestivum* | GAV | CP, MP |
| KP341643 | Qinghai | 28-Jul-2011 | *Triticum aestivum* | GAV | CP, MP |
| KP341644 | Qinghai | 28-Jul-2011 | *Triticum aestivum* | GAV | CP, MP |
| EU386786*^b^* | Ningxia | 2005 | *Triticum aestivum* | GAV | CP, MP |
| EU386795*^b^* | Ningxia | 03-Jun-2005 | *Triticum aestivum* | GAV | CP, MP |
| EU386798*^b^* | Ningxia | 31-May-2004 | *Triticum aestivum* | GAV | MP |
| EU386805*^b^* | Ningxia | 31-May-2004 | *Triticum aestivum* | GAV | CP, MP |
| KP096611 | Ningxia | 01-Jun-2007 | *Triticum aestivum* | PAV | CP, MP |
| KP341555 | Ningxia | 02-Jun-2007 | *Triticum aestivum* | GAV | CP, MP |
| KP341556 | Ningxia | 02-Jun-2007 | *Triticum aestivum* | GAV | CP, MP |
| KP341557 | Ningxia | 02-Jun-2007 | *Triticum aestivum* | GAV | CP, MP |
| KP341558 | Ningxia | 02-Jun-2007 | *Triticum aestivum* | GAV | CP, MP |
| KP341560 | Ningxia | 01-Jun-2007 | *Triticum aestivum* | GAV | CP, MP |
| KP341561 | Ningxia | 01-Jun-2007 | *Triticum aestivum* | GAV | CP, MP |
| EU332328*^a^* | Guizhou | 2006 | *Triticum aestivum* | PAV | CP, MP |
| EU332329*^a^* | Guizhou | 2006 | *Triticum aestivum* | PAV | MP |
| KP096601 | Guizhou | 12-Mar-2007 | *Triticum aestivum* | PAV | CP, MP |
| KP096639 | Guizhou | 13-Mar-2009 | *Triticum aestivum* | PAV | MP |
| KP096640 | Guizhou | 13-Mar-2009 | *Triticum aestivum* | PAV | CP, MP |
| KP096642 | Guizhou | 13-Mar-2009 | *Triticum aestivum* | PAV | MP |
| KP096643 | Guizhou | 13-Mar-2009 | *Triticum aestivum* | PAV | MP |
| KP096693 | Guizhou | 01-Mar-2006 | *Triticum aestivum* | PAV | CP, MP |
| KP096694 | Guizhou | 01-Mar-2006 | *Triticum aestivum* | PAV | CP, MP |
| KP341547 | Guizhou | 12-Mar-2007 | *Triticum aestivum* | GAV | CP, MP |
| KP341652 | Guizhou | 13-Mar-2009 | *Triticum aestivum* | GAV | CP, MP |
| KP096614 | Sichuan | 26-Mar-2007 | *Triticum aestivum* | PAV | CP, MP |
| KP096613 | Sichuan | 26-Mar-2007 | *Triticum aestivum* | PAV | CP, MP |
| KP096630 | Sichuan | 21-Mar-2008 | *Triticum aestivum* | PAV | CP, MP |
| KP096631 | Sichuan | 21-Mar-2008 | *Triticum aestivum* | PAV | MP |
| KP096648 | Sichuan | 12-Mar-2009 | *Triticum aestivum* | PAV | MP |
| KP096652 | Sichuan | 21-Mar-2010 | *Triticum aestivum* | PAV | CP, MP |
| KP096653 | Sichuan | 21-Mar-2010 | *Triticum aestivum* | PAV | CP, MP |
| KP096654 | Sichuan | 21-Mar-2010 | *Triticum aestivum* | PAV | CP, MP |
| KP096655 | Sichuan | 21-Mar-2010 | *Triticum aestivum* | PAV | CP, MP |
| KP096660 | Sichuan | 19-Mar-2011 | *Triticum aestivum* | PAV | CP, MP |
| KP096661 | Sichuan | 19-Mar-2011 | *Triticum aestivum* | PAV | CP, MP |
| MN648439 | Sichuan | 09-Apr-2018 | *Triticum aestivum* | PAV | CP, MP |
| OK075102 | Sichuan | 01-Apr-2021 | *Triticum aestivum* | PAV | CP, MP |
| OK075105 | Sichuan | 01-Apr-2021 | *Triticum aestivum* | PAV | CP, MP |
| OK075107 | Sichuan | 01-Apr-2021 | *Triticum aestivum* | PAV | CP, MP |
| OK075106 | Sichuan | 01-Apr-2021 | *Triticum aestivum* | PAV | CP, MP |
| OK075108 | Sichuan | 01-Apr-2021 | *Triticum aestivum* | PAV | CP, MP |
| OK075104 | Sichuan | 01-Apr-2021 | *Triticum aestivum* | PAV | CP, MP |
| OK075103 | Sichuan | 01-Apr-2021 | *Triticum aestivum* | PAV | CP, MP |
| EU332332*^a^* | Yunnan | 2006 | *Triticum aestivum* | PAV | CP, MP |
| EU332333*^a^* | Yunnan | 2006 | *Avena sativa* | PAV | CP, MP |
| EU386811*^a^* | Yunnan | 2006 | unknown | GAV | CP, MP |
| EU386814*^a^* | Yunnan | 2006 | unknown | GAV | CP, MP |
| EU386815*^a^* | Yunnan | 2006 | unknown | GAV | CP, MP |
| KP096619 | Yunnan | 11-Mar-2007 | *Triticum aestivum* | PAV | CP, MP |
| KP096620 | Yunnan | 11-Mar-2007 | *Triticum aestivum* | PAV | CP, MP |
| KP096657 | Yunnan | 20-Jun-2010 | *Triticum aestivum* | PAV | CP, MP |
| KP096667 | Yunnan | 19-Mar-2011 | *Triticum aestivum* | PAV | CP, MP |
| KP096671 | Yunnan | 19-Mar-2011 | *Triticum aestivum* | PAV | CP, MP |
| KP096672 | Yunnan | 19-Mar-2011 | *Triticum aestivum* | PAV | CP, MP |
| KP341576 | Yunnan | 11-Mar-2007 | *Triticum aestivum* | GAV | CP, MP |
| KP341577 | Yunnan | 11-Mar-2007 | *Triticum aestivum* | GAV | CP, MP |
| KP341586 | Yunnan | 21-Mar-2008 | *Triticum aestivum* | GAV | CP, MP |
| KP341587 | Yunnan | 21-Mar-2008 | *Triticum aestivum* | GAV | CP, MP |
| KP341602 | Yunnan | 11-Mar-2009 | *Triticum aestivum* | GAV | CP, MP |
| KP341603 | Yunnan | 11-Mar-2009 | *Triticum aestivum* | GAV | CP, MP |
| KP341604 | Yunnan | 11-Mar-2009 | *Triticum aestivum* | GAV | CP, MP |
| KP341606 | Yunnan | 11-Mar-2009 | *Triticum aestivum* | GAV | CP, MP |
| MN648421 | Yunnan | 18-Feb-2017 | *Hordeum vulgare* | PAV | CP, MP |
| MN648422 | Yunnan | 06-Mar-2018 | *Triticum aestivum* | PAV | CP, MP |
| MN648423 | Yunnan | 24-Mar-2019 | *Triticum aestivum* | PAV | CP, MP |
| AY855920 | unknow | 1996 | *Triticum aestivum* | PAV | CP, MP |
| EU386773 | Liaoning | 2006 | unknown | GAV | MP |
| EU386776*^b^* | unknow | 2004 | *Triticum aestivum* | GAV | CP |
| KP096596 | Xinjiang | 05-Jun-2007 | *Triticum aestivum* | PAV | CP, MP |
| KP096598 | Xinjiang | 05-Jun-2007 | *Triticum aestivum* | PAV | CP, MP |
| KP341584 | Tibet | 25-Jun-2008 | *Triticum aestivum* | GAV | CP, MP |
| KP341585 | Tibet | 25-Jun-2008 | *Triticum aestivum* | GAV | CP, MP |
| KY634899 | unknow | 2011 | *Hordeum vulgare* | PAV | CP, MP |
| MN648425 | Shaanxi | 18-Apr-2018 | *Triticum aestivum* | PAV | MP |
| MW690138 | unknow | 2018 | *Hordeum vulgare* | GAV | CP, MP |
| AF216863 | unknow | unknown | unknown | GPV | CP, MP |
| EF153722*^b^* | Gansu | 2004 | *Triticum aestivum* | GPV | CP, MP |
| EF153723*^b^* | Shaanxi | 2004 | *Triticum aestivum* | GPV | CP, MP |
| EF174408*^b^* | Shanxi | 2004 | *Triticum aestivum* | GPV | CP, MP |
| EF174409*^b^* | Shanxi | 2004 | *Triticum aestivum* | GPV | CP, MP |
| EF174410*^b^* | Shanxi | 2004 | *Triticum aestivum* | GPV | CP, MP |
| EF174411*^b^* | Shanxi | 2004 | *Triticum aestivum* | GPV | CP, MP |
| EF174412*^b^* | Gansu | 2005 | *Triticum aestivum* | GPV | CP, MP |
| EF174413*^b^* | Shanxi | 2005 | *Triticum aestivum* | GPV | CP, MP |
| EF174415*^b^* | Shanxi | 2005 | *Triticum aestivum* | GPV | CP, MP |
| EF174416*^a^* | Henan | 2006 | *Triticum aestivum* | GPV | CP, MP |
| KP096553 | Gansu | 14-Apr-2007 | *Triticum aestivum* | GPV | CP, MP |
| KP096554 | Guizhou | 12-Mar-2007 | *Triticum aestivum* | GPV | CP, MP |
| KP096555 | Guizhou | 12-Mar-2007 | *Triticum aestivum* | GPV | CP, MP |
| KP096556 | Henan | 05-Apr-2007 | *Triticum aestivum* | GPV | CP, MP |
| KP096557 | Henan | 07-Apr-2007 | *Triticum aestivum* | GPV | CP, MP |
| KP096558 | Shandong | 27-Apr-2007 | *Triticum aestivum* | GPV | CP, MP |
| KP096559 | Shandong | 27-Apr-2007 | *Triticum aestivum* | GPV | CP, MP |
| KP096560 | Shaanxi | 16-Apr-2007 | *Triticum aestivum* | GPV | CP, MP |
| KP096561 | Shanxi | 26-Apr-2007 | *Triticum aestivum* | GPV | CP, MP |
| KP096562 | Shanxi | 26-Apr-2007 | *Triticum aestivum* | GPV | CP, MP |
| KP096563 | Shanxi | 26-Apr-2007 | *Triticum aestivum* | GPV | CP, MP |
| KP096565 | Shaanxi | 15-Apr-2007 | *Triticum aestivum* | GPV | CP, MP |
| KP096566 | Shanxi | 23-Apr-2008 | *Triticum aestivum* | GPV | CP, MP |
| KP096567 | Shanxi | 23-Apr-2008 | *Triticum aestivum* | GPV | CP, MP |
| KP096569 | Gansu | 23-May-2009 | *Triticum aestivum* | GPV | CP, MP |
| KP096570 | Gansu | 23-May-2009 | *Triticum aestivum* | GPV | CP, MP |
| KP096571 | Gansu | 22-May-2009 | *Triticum aestivum* | GPV | CP, MP |
| KP096572 | Gansu | 22-May-2009 | *Triticum aestivum* | GPV | CP, MP |
| KP096573 | Gansu | 22-May-2009 | *Triticum aestivum* | GPV | CP, MP |
| KP096574 | Henan | 20-Mar-2009 | *Triticum aestivum* | GPV | CP, MP |
| KP096576 | Shaanxi | 17-Apr-2009 | *Triticum aestivum* | GPV | CP, MP |
| KP096577 | Shaanxi | 17-Apr-2009 | *Triticum aestivum* | GPV | CP, MP |
| KP096581 | Shaanxi | 17-Apr-2009 | *Triticum aestivum* | GPV | CP, MP |
| KP096582 | Shanxi | 27-Apr-2009 | *Triticum aestivum* | GPV | CP, MP |
| KP096584 | Shanxi | 28-Apr-2009 | *Triticum aestivum* | GPV | CP, MP |
| KP096588 | Shanxi | 17-May-2011 | *Triticum aestivum* | GPV | CP, MP |
| KP096590 | Shanxi | 18-May-2011 | *Triticum aestivum* | GPV | CP, MP |
| KP096591 | Shanxi | 19-May-2010 | *Triticum aestivum* | GPV | CP, MP |
| KP096592 | Shanxi | 18-May-2011 | *Triticum aestivum* | GPV | CP, MP |
| L10356 | unknown | unknown | unknown | GPV | CP, MP |
| NC039035 | Shanxi | unknown | *Triticum aestivum* | GPV | CP, MP |
| KY634910 | Czech Republic | 2015 | *Hordeum vulgare* | PAV | CP, MP |
| KY634911 | Czech Republic | 2015 | *Hordeum vulgare* | PAV | CP, MP |
| MK012643 | Estonia | 11-Jul-2012 | *Triticum aestivum* | OYV | CP, MP |
| MK012644 | Estonia | 02-Jul-2013 | *Avena sativa* | OYV | CP, MP |
| MK012645 | Estonia | 02-Jul-2013 | *Triticum aestivum* | OYV | CP, MP |
| MK012646 | Estonia | 02-Jul-2013 | *Avena sativa* | OYV | CP |
| MK012647 | Estonia | 02-Jul-2013 | *Avena sativa* | OYV | CP, MP |
| MK012648 | Estonia | 28-Jun-2013 | *Avena sativa* | OYV | CP, MP |
| MK012649 | Estonia | 23-Jul-2014 | *Avena sativa* | OYV | CP, MP |
| MK012650 | Estonia | 28-Jun-2013 | *Triticum aestivum* | PAS | MP |
| MK012651 | Estonia | 23-Jul-2014 | *Triticum aestivum* | PAS | MP |
| MK012652 | Estonia | 23-Jul-2014 | *Triticum aestivum* | PAS | MP |
| MK012653 | Estonia | 23-Jul-2014 | *Hordeum vulgare* | PAS | CP, MP |
| MK012654 | Estonia | 18-Jul-2014 | *Triticum aestivum* | PAS | MP |
| MK012655 | Estonia | 17-Jun-2014 | *Triticum aestivum* | PAS | MP |
| MK012656 | Estonia | 14-Jul-2015 | *Triticum aestivum* | PAS | MP |
| MK012657 | Estonia | 29-Jun-2015 | *Triticum aestivum* | PAS | CP, MP |
| MK012658 | Estonia | 02-Jun-2015 | *Triticum aestivum* | PAS | MP |
| MK012659 | Estonia | 18-Jul-2015 | *Triticum aestivum* | PAS | MP |
| MK012660 | Estonia | 25-May-2015 | *Secale cereale* | PAS | MP |
| MK012661 | Estonia | 23-Jul-2014 | *Hordeum vulgare* | OYV | CP, MP |
| MK012662 | Estonia | 18-Jul-2014 | *Triticum aestivum* | GAV | MP |
| MK012663 | Estonia | 18-Aug-2015 | *Avena sativa* | GAV | MP |
| AY040343 | France | 1985 | *Hordeum vulgare* | PAV | CP, MP |
| AY040344 | France | 1996 | *Hordeum vulgare* | PAV | CP, MP |
| KY634879 | Germany | 2008 | *Lolium multiflorum* | PAV | MP |
| KY634880 | Germany | 2015 | *Secale cereale* | PAV | CP, MP |
| KY634881 | Germany | 2015 | *Lolium multiflorum* | PAV | CP, MP |
| KY634882 | Germany | 2008 | *Lolium multiflorum* | PAV | CP, MP |
| KY634883 | Germany | 2008 | *Lolium multiflorum* | PAV | CP, MP |
| KY634884 | Germany | 2008 | *Festuca ovina* | PAV | CP, MP |
| KY634888 | Germany | 2015 | *Hordeum vulgare* | PAV | MP |
| KY634890 | Germany | 2015 | *Hordeum vulgare* | PAV | CP, MP |
| KY634891 | Germany | 2015 | *Hordeum vulgare* | PAV | MP |
| KY634893 | Germany | 2015 | unknown | PAV | CP, MP |
| KY634894 | Germany | 2015 | *Triticum aestivum* | PAV | CP, MP |
| KY634895 | Germany | 2015 | *Triticum aestivum* | PAV | CP, MP |
| KY634896 | Germany | 2015 | *Triticum aestivum* | PAV | CP, MP |
| KY634907 | Germany | 2015 | *Hordeum vulgare* | PAV | MP |
| KY634908 | Germany | 2015 | *Zea mays* | PAV | MP |
| KY634909 | Germany | 2015 | *Zea mays* | PAV | MP |
| KY634913 | Germany | 2015 | *Triticum aestivum* | PAV | MP |
| KY634915 | Germany | 2008 | *Festuca ovina* | PAV | CP, MP |
| KY634916 | Germany | 2008 | *Festuca ovina* | PAV | MP |
| KY634917 | Germany | 2008 | *Festuca ovina* | PAV | MP |
| KY634922 | Germany | 2016 | *Hordeum vulgare* | PAV | MP |
| KY634925 | Germany | 2015 | *Hordeum vulgare* | PAV | CP, MP |
| KP096696 | Hungary | 01-May-2010 | *Triticum aestivum* | PAV | CP, MP |
| KP771878 | Iran | 2010 | *Hordeum vulgare* | PAV | CP, MP |
| KY634886 | Netherlands | 2015 | *Lolium perenne* | PAV | CP, MP |
| KY634918 | Netherlands | 2016 | *Zea mays* | PAV | CP, MP |
| EF408152 | New Zealand | 2003-2004 | *Microlaena stipoides* | PAS | CP, MP |
| EF408154 | New Zealand | 2003-2004 | *Dichelachne crinita* | PAS | CP, MP |
| EF408155 | New Zealand | 2006 | *Triticum aestivum* | PAS | CP, MP |
| EF408156 | New Zealand | 2003-2004 | *Microlaena stipoides* | PAV | CP, MP |
| EF408157 | New Zealand | 2003-2004 | *Microlaena stipoides* | PAV | CP, MP |
| EF408158 | New Zealand | 2003-2004 | *Microlaena stipoides* | PAV | CP, MP |
| EF408159 | New Zealand | 2003-2004 | *Microlaena stipoides* | PAV | CP, MP |
| EF408160 | New Zealand | 2003-2004 | *Microlaena stipoides* | PAV | CP, MP |
| EF408162 | New Zealand | 2003-2004 | *Microlaena stipoides* | PAV | CP, MP |
| EF408164 | New Zealand | 2003-2004 | *Microlaena stipoides* | PAV | CP, MP |
| EF408165 | New Zealand | 2003-2004 | *Microlaena stipoides* | PAV | CP, MP |
| EF408168 | New Zealand | 2003-2004 | *Anthoxanthum redolens* | PAV | CP, MP |
| EF408169 | New Zealand | 2003-2004 | *Microlaena stipoides* | PAV | CP, MP |
| EF408170 | New Zealand | 2003-2004 | *Festuca novae-zelandiae* | PAV | CP, MP |
| EF408171 | New Zealand | 2003-2004 | *Festuca novae-zelandiae* | PAV | CP, MP |
| EF408172 | New Zealand | 2003-2004 | *Dichelachne crinita* | PAV | CP, MP |
| EF408173 | New Zealand | 2005-2006 | *Hordeum vulgare* | PAV | MP |
| EF408174 | New Zealand | 2005-2006 | *Triticum aestivum* | PAV | MP |
| EF408175 | New Zealand | 2005-2006 | *Avena sativa* | PAV | MP |
| EF408176 | New Zealand | 2003-2004 | *Poa cita* | PAV | MP |
| EF408177 | New Zealand | 2005-2006 | *Avena sativa* | PAV | MP |
| EF408179 | New Zealand | 2003-2004 | *Microlaena stipoides* | PAV | MP |
| EF408180 | New Zealand | 2005-2006 | *Avena sativa* | MAV | CP, MP |
| EF408181 | New Zealand | 2005-2006 | *Festuca novae-zelandiae* | MAV | CP, MP |
| EF408182 | New Zealand | 2005-2006 | *Triticum aestivum* | MAV | MP |
| EF408183 | New Zealand | 2005-2006 | *Festuca novae-zelandiae* | MAV | MP |
| EF408184 | New Zealand | 2005-2006 | *Festuca novae-zelandiae* | MAV | MP |
| EF408185 | New Zealand | 2003-2004 | *Anthoxanthum redolens* | MAV | CP, MP |
| KY634900 | New Zealand | 2000 | *Hordeum vulgare* | PAV | CP, MP |
| GU247975 | Pakistan | Mar-2008 | *Triticum aestivum* | PAV | MP |
| GU247976 | Pakistan | Mar-2008 | *Triticum aestivum* | PAV | MP |
| GU247977 | Pakistan | Mar-2008 | *Triticum aestivum* | PAV | MP |
| GU247978 | Pakistan | Mar-2008 | *Triticum aestivum* | PAV | MP |
| GU247979 | Pakistan | Mar-2008 | *Triticum aestivum* | PAV | MP |
| GU247980 | Pakistan | Mar-2008 | *Triticum aestivum* | PAV | MP |
| GU247981 | Pakistan | Mar-2008 | *Triticum aestivum* | PAV | MP |
| GU247982 | Pakistan | Mar-2008 | *Triticum aestivum* | PAV | MP |
| HE584722 | Pakistan | 09-Jan-2011 | *Avena sativa* | PAV | CP, MP |
| HE985229 | Pakistan | 22-Jan-2011 | *Triticum aestivum* | PAV | CP, MP |
| JQ811488 | Pakistan | 04-Apr-2011 | *Avena sativa* | PAV | CP, MP |
| JQ811489 | Pakistan | 02-Oct-2011 | *Zea mays* | PAV | CP, MP |
| JX473287 | Pakistan | 27-Nov-2011 | *Sorghum halepense* | PAV | MP |
| JX473288 | Pakistan | 27-Nov-2011 | *Lolium multiflorum* | PAV | CP, MP |
| KR259156 | Pakistan | 17-Oct-2011 | *Pennisetum glaucum* | PAS | CP, MP |
| KR259157 | Pakistan | 17-Oct-2011 | *Pennisetum glaucum* | PAV | CP, MP |
| KT198975 | Pakistan | 17-Feb-2011 | *Triticum aestivum* | PAV | CP, MP |
| KT198976 | Pakistan | 17-Feb-2011 | *Triticum aestivum* | PAV | CP, MP |
| KT198978 | Pakistan | 24-Jun-2012 | *Zea mays* | PAV | CP, MP |
| KT198982 | Pakistan | 05-Apr-2011 | *Hordeum vulgare* | PAV | CP, MP |
| KT198983 | Pakistan | 05-Apr-2011 | *Triticum aestivum* | PAV | CP, MP |
| KT198984 | Pakistan | 23-Feb-2011 | *Triticum aestivum* | PAV | CP, MP |
| KT198985 | Pakistan | 23-Feb-2011 | *Triticum aestivum* | PAV | CP, MP |
| KT222666 | Pakistan | 21-Feb-2013 | *Triticum aestivum* | PAV | MP |
| KT222667 | Pakistan | 21-Feb-2013 | *Triticum aestivum* | PAV | MP |
| KT222668 | Pakistan | 21-Feb-2013 | *Triticum aestivum* | PAV | MP |
| KT222669 | Pakistan | 21-Feb-2013 | *Triticum aestivum* | PAV | MP |
| KT222670 | Pakistan | 21-Feb-2013 | *Triticum aestivum* | PAV | MP |
| KT222671 | Pakistan | 21-Feb-2013 | *Triticum aestivum* | PAV | MP |
| KT222672 | Pakistan | 21-Feb-2013 | *Triticum aestivum* | PAV | MP |
| KT252975 | Pakistan | 21-Feb-2013 | *Triticum aestivum* | PAV | CP, MP |
| KT252976 | Pakistan | 21-Feb-2013 | *Triticum aestivum* | PAV | CP, MP |
| KT252977 | Pakistan | 21-Feb-2013 | *Triticum aestivum* | PAV | CP, MP |
| KT252978 | Pakistan | 21-Feb-2013 | *Triticum aestivum* | PAV | CP, MP |
| KU893149 | Poland | Apr-2015 | *Hordeum vulgare* | MAV | MP |
| KU097016 | South Korea | Feb-2014 | *Avena sativa* | PAV | CP, MP |
| LC530629 | South Korea | 30-Mar-2019 | *Avena sativa* | PAV | CP, MP |
| LC530630 | South Korea | 20-Apr-2019 | *Avena sativa* | PAV | CP, MP |
| LC550011 | South Korea | 03-Apr-2020 | *Avena sativa* | PAV | CP, MP |
| LC550012 | South Korea | 03-Apr-2020 | *Avena sativa* | PAV | CP, MP |
| LC550014 | South Korea | 24-Apr-2020 | *Avena sativa* | PAV | CP, MP |
| LC550013 | South Korea | 15-May-2020 | *Avena sativa* | PAV | CP, MP |
| LC550015 | South Korea | 24-Apr-2020 | *Avena sativa* | PAV | CP, MP |
| LC550016 | South Korea | 24-Apr-2020 | *Avena sativa* | PAV | CP, MP |
| LC550017 | South Korea | 03-Apr-2020 | *Avena sativa* | PAV | MP |
| LC586074 | South Korea | 24-Apr-2020 | *Rhopalosiphum padi* | PAV | MP |
| LC586075 | South Korea | 24-Apr-2020 | *Rhopalosiphum padi* | PAV | CP, MP |
| LC586076 | South Korea | 24-Apr-2020 | *Sitobion avenae* | PAV | MP |
| LC586077 | South Korea | 14-May-2020 | *Rhopalosiphum padi* | PAV | MP |
| LC586078 | South Korea | 14-May-2020 | *Rhopalosiphum padi* | PAV | MP |
| LC637413 | South Korea | 06-May-2021 | *Hordeum vulgare* | PAV | CP, MP |
| LC628200 | South Korea | 29-Mar-2021 | *Avena sativa* | PAV | CP, MP |
| LC628201 | South Korea | 29-Mar-2021 | *Hordeum vulgare* | PAV | CP, MP |
| LC628202 | South Korea | 08-Apr-2021 | *Triticum aestivum* | PAV | CP, MP |
| LC631803 | South Korea | 22-Apr-2021 | *Hordeum vulgare* | PAV | CP, MP |
| LC631804 | South Korea | 29-Apr-2021 | *Sitobion avenae* | PAV | MP |
| LC637409 | South Korea | 26-May-2021 | *Triticum aestivum* | PAV | CP, MP |
| LC637408 | South Korea | 12-May-2021 | *Triticum aestivum* | PAV | CP, MP |
| LC637414 | South Korea | 19-May-2021 | *Hordeum vulgare* | PAV | CP, MP |
| LC637417 | South Korea | 06-May-2021 | *Triticum aestivum* | PAV | CP, MP |
| LC637410 | South Korea | 12-May-2021 | *Hordeum vulgare* | PAV | CP, MP |
| LC637411 | South Korea | 26-May-2021 | *Triticum aestivum* | PAV | CP, MP |
| LC637412 | South Korea | 27-May-2021 | *Triticum aestivum* | PAV | CP, MP |
| LC639193 | South Korea | 29-Mar-2021 | *Rhopalosiphum padi* | PAV | CP, MP |
| LC639195 | South Korea | 08-Apr-2021 | *Sitobion avenae* | PAV | CP, MP |
| LC639196 | South Korea | 22-Apr-2021 | *Rrhopalosiphum maidis* | PAV | CP, MP |
| MF693123 | South Korea | 01-Mar-2015 | *Hordeum vulgare* | PAV | CP, MP |
| MF693124 | South Korea | 01-Mar-2015 | *Hordeum vulgare* | PAV | CP, MP |
| MF693130 | South Korea | 01-Mar-2015 | *Hordeum vulgare* | PAV | CP, MP |
| MF693133 | South Korea | 01-Mar-2015 | *Hordeum vulgare* | PAV | CP, MP |
| KY634903 | Sweden | 2000 | *Hordeum vulgare* | PAV | CP, MP |
| MK012642 | Sweden | Jun-2010 | *Festuca pratensis* | PAV | CP, MP |
| MN493946 | Sweden | 2012 | *Lolium perenne* | PAV | MP |
| MK224495 | Tunisia | 2018 | *x Triticosecale* | MAV | MP |
| MK224496 | Tunisia | 2018 | *x Triticosecale* | MAV | MP |
| KC900900 | Turkey | 20-Jul-2012 | *Triticum aestivum* | PAV | CP, MP |
| KX774424 | Turkey | 10-May-2016 | *Triticum aestivum* | PAV | CP, MP |
| MK732034 | Turkey | 16-May-2017 | *Triticum aestivum* | PAV | CP, MP |
| DQ631840 | USA | 2002 | *Danthonia californica* | MAV | MP |
| DQ631841 | USA | 2002 | *Danthonia californica* | MAV | MP |
| DQ631842 | USA | 2003 | *Festuca californica* | MAV | MP |
| DQ631843 | USA | 2002 | *Festuca californica* | MAV | MP |
| DQ631847 | USA | 2002 | *Elymus multisetus* | PAV | CP, MP |
| DQ631848 | USA | 2002 | *Avena fatua* | PAV | CP, MP |
| DQ631849 | USA | 2002 | *Avena fatua* | PAV | CP, MP |
| DQ631850 | USA | 2002 | *Avena fatua* | PAV | CP, MP |
| DQ631851 | USA | 2002 | *Avena fatua* | PAV | CP, MP |
| DQ631852 | USA | 2002 | *Avena fatua* | PAV | MP |
| DQ631853 | USA | 2002 | *Avena fatua* | PAV | CP, MP |
| DQ631854 | USA | 2002 | *Elymus multisetus* | PAV | CP, MP |
| DQ631855 | USA | 2002 | *Elymus multisetus* | PAV | CP, MP |
| DQ631856 | USA | 2002 | *Phalaris coerulescens* | PAV | CP, MP |
| DQ631857 | USA | 2002 | *Glyceria striata* | PAV | CP, MP |
| DQ680114 | USA | 2003 | *Avena sativa* | MAV | MP |
| DQ680115 | USA | 2003 | *Avena sativa* | MAV | MP |
| DQ680116 | USA | 2003 | *Avena sativa* | MAV | MP |
| DQ680117 | USA | 2003 | *Avena sativa* | MAV | MP |
| DQ680118 | USA | 2003 | *Avena sativa* | MAV | MP |
| DQ680119 | USA | 2003 | *Avena sativa* | MAV | MP |
| DQ680120 | USA | 2003 | *Avena sativa* | MAV | MP |
| DQ680121 | USA | 2003 | *Avena sativa* | MAV | MP |
| DQ680122 | USA | 2003 | *Avena sativa* | MAV | MP |
| DQ680123 | USA | 2003 | *Avena sativa* | MAV | MP |
| DQ680125 | USA | 2003 | *Avena sativa* | MAV | MP |
| DQ680126 | USA | 2003 | *Avena sativa* | MAV | MP |
| DQ680129 | USA | 2003 | *Avena sativa* | MAV | MP |
| DQ680130 | USA | 2003 | *Avena sativa* | MAV | MP |
| DQ680131 | USA | 2003 | *Avena sativa* | MAV | MP |
| DQ680133 | USA | 2003 | *Avena sativa* | MAV | MP |
| DQ680134 | USA | 2003 | *Avena sativa* | MAV | MP |
| DQ680135 | USA | 2003 | *Avena sativa* | MAV | MP |
| DQ792506 | USA | 2003 | *Avena sativa* | PAS | MP |
| KU170668 | USA | 02-Apr-2011 | *Triticum aestivum* | PAV | MP |
| KY593456 | USA | 09-May-2011 | unknown | PAS | CP, MP |
| KY593457 | USA | 07-May-2012 | *Triticum aestivum* | PAS | MP |
| KY593458 | USA | 09-May-2011 | *Triticum aestivum* | PAV | CP, MP |
| KY621333 | USA | 2011 | *Triticum aestivum* | PAV | CP, MP |
| MK913611 | USA | Mar-2016 | *Triticum aestivum* | PAV | CP, MP |
| MK913612 | USA | Mar-2016 | *Triticum aestivum* | PAV | CP, MP |
| MK913613 | USA | Mar-2016 | *Triticum aestivum* | PAV | CP, MP |
| MK913614 | USA | Mar-2016 | *Triticum aestivum* | PAS | CP, MP |
| MK913615 | USA | Mar-2016 | *Triticum aestivum* | PAS | CP, MP |
| MK913616 | USA | Mar-2016 | *Triticum aestivum* | PAS | CP, MP |
| MK913617 | USA | Mar-2016 | *Triticum aestivum* | PAS | CP, MP |
| MK913618 | USA | Mar-2016 | *Triticum aestivum* | PAS | CP, MP |
| MK913619 | USA | Mar-2016 | *Triticum aestivum* | PAS | CP, MP |
| MK913620 | USA | Mar-2016 | *Triticum aestivum* | PAS | MP |
| MN128939 | USA | Mar-2016 | *Triticum aestivum* | PAS | CP |
| MN128940 | USA | Mar-2016 | *Triticum aestivum* | PAS | MP |
| MN128941 | USA | Mar-2016 | *Triticum aestivum* | PAV | CP, MP |
| AY540130 | USA | unknown | unknown | SGV | CP, MP |
| AY541037 | USA | unknown | unknown | SGV | CP, MP |
| AY541038 | USA | unknown | unknown | SGV | CP, MP |
| NC043124 | USA | unknown | unknown | SGV | CP, MP |
| U06865 | USA | unknown | *Hordeum vulgare* | SGV | CP, MP |

*^a^* Liu, S. Q. 2008. Specific Detection of Barley Yellow Dwarf Viruses and Population Genetic Variation of BYDV PAV Species. Hunan Agricultural University Master Dissertation.

*^b^* Sun, B. 2006. Molecular variability of Barley yellow dwarf viruses. Chinese Academy of Agricultural Sciences Master Dissertation.

Supplementary Table. 2 Marginal likelihoods of different combinations of clock models and tree priors.

| Genes | Datasets | Model of rate variation | Coalescent tree prior | Log marginal likelihood |
| --- | --- | --- | --- | --- |
| Coat protein | 379 | Strict clock | Bayesian skyline | -11063.168 |
|  |  | Strict clock | Constant size | -11109.125 |
|  |  | Strict clock | Exponential growth | -11110.084 |
|  |  | **Uncorrelated lognormal relaxed clock** | **Bayesian skyline** | **-11043.760** |
|  |  | Uncorrelated lognormal relaxed clock | Constant size | -11087.639 |
|  |  | Uncorrelated lognormal relaxed clock | Exponential growth | -11072.410 |
|  | 356 | Strict clock | Bayesian skyline | -10450.167 |
|  |  | Strict clock | Constant size | -10467.402 |
|  |  | Strict clock | Exponential growth | -10470.380 |
|  |  | **Uncorrelated lognormal relaxed clock** | **Bayesian skyline** | **-10433.093** |
|  |  | Uncorrelated lognormal relaxed clock | Constant size | -10452.644 |
|  |  | Uncorrelated lognormal relaxed clock | Exponential growth | -10447.582 |
|  | 333 | Strict clock | Bayesian skyline | -9672.916 |
|  |  | Strict clock | Constant size | -9696.274 |
|  |  | Strict clock | Exponential growth | -9702.012 |
|  |  | **Uncorrelated lognormal relaxed clock** | **Bayesian skyline** | **-9644.971** |
|  |  | Uncorrelated lognormal relaxed clock | Constant size | -9688.130 |
|  |  | Uncorrelated lognormal relaxed clock | Exponential growth | -9692.846 |
| Movement  protein | 485 | Strict clock | Bayesian skyline | -8442.872 |
|  |  | Strict clock | Constant size | -8474.064 |
|  |  | Strict clock | Exponential growth | -8472.643 |
|  |  | **Uncorrelated lognormal relaxed clock** | **Bayesian skyline** | **-8407.652** |
|  |  | Uncorrelated lognormal relaxed clock | Constant size | -8427.965 |
|  |  | Uncorrelated lognormal relaxed clock | Exponential growth | -8437.547 |
|  | 458 | Strict clock | Bayesian skyline | -7916.399 |
|  |  | Strict clock | Constant size | -7930.732 |
|  |  | Strict clock | Exponential growth | -7918.638 |
|  |  | **Uncorrelated lognormal relaxed clock** | **Bayesian skyline** | **-7892.592** |
|  |  | Uncorrelated lognormal relaxed clock | Constant size | -7912.467 |
|  |  | Uncorrelated lognormal relaxed clock | Exponential growth | -7922.079 |
|  | 415 | Strict clock | Bayesian skyline | -7452.441 |
|  |  | Strict clock | Constant size | -7471.954 |
|  |  | Strict clock | Exponential growth | -7468.131 |
|  |  | Uncorrelated lognormal relaxed clock | Bayesian skyline | -7425.941 |
|  |  | Uncorrelated lognormal relaxed clock | Constant size | -7460.688 |
|  |  | Uncorrelated lognormal relaxed clock | Exponential growth | -7472.909 |

The best-fitting tree prior and molecular clock model are indicated in bold font.

Supplementary Table. 3 Timescale analysis of BYDV using different datesets by cp and mp gene.

| Genes | Date sets | Methods | MRCA*^a^* (Year) | Substitution rate (subs/site/year) |
| --- | --- | --- | --- | --- |
| Coat protein | 379 | Bayesian | 1433.9 (1039.5–1766.1) | 8.327×10^-4^ (4.700×10^-4^–1.228×10^-3^) |
|  |  | Bayesian (with discrete location states) | 1723.4 (1559.0–1867.0) | 1.240×10^-3^ (8.401×10^-4^–1.669×10^-3^) |
|  |  | Maximum likelihood | 1547.1 | 9.927×10^-4^ |
|  | 356 | Bayesian | 1715.3 (1512.3–1877.6) | 1.491×10^-3^ (9.364×10^-4^–2.065×10^-3^) |
|  |  | Bayesian (with discrete location states) | 1870.2 (1801.1–1930.8) | 1.924×10^-3^ (1.441×10^-3^–2.421×10^-3^) |
|  |  | Maximum likelihood | 1731.1 | 1.483×10^-3^ |
|  | 333 | Bayesian | 1407.4 (969.3–1735.7) | 6.550×10^-4^ (4.313×10^-4^–8.977×10^-4^) |
|  |  | Bayesian (with discrete location states) | 1599.8 (1328.2–1811.8) | 7.695×10^-4^ (4.957×10^-4^–1.070×10^-3^) |
|  |  | Maximum likelihood | 1770.1 | 1.888×10^-3^ |
|  | 308 | Bayesian | 782.5 (37.1–1473.0) | 3.811×10^-4^ (2.027×10^-4^–5.692×10^-4^) |
|  |  | Bayesian (with discrete location states) | 1332.8 (928.4–1701.0) | 5.359×10^-4^ (3.330×10^-4^–7.828×10^-4^) |
|  |  | Maximum likelihood | 1400.6 | 6.244×10^-4^ |
|  | 283 | Bayesian | 1123.6 (608.7–1565.9) | 4.962×10^-4^ (3.000×10^-4^–7.189×10^-4^) |
|  |  | Bayesian (with discrete location states) | 1364.2 (1015.8–1663.9) | 5.773×10^-4^ (3.704×10^-4^–8.011×10^-4^) |
|  |  | Maximum likelihood | 1640.4 | 1.028×10^-3^ |
|  | 258 | Bayesian | 989.5 (430.8–1516.1) | 4.135×10^-4^ (2.617×10^-4^–6.383×10^-4^) |
|  |  | Bayesian (with discrete location states) | 1357.9 (906.3.0–1734.6) | 5.692×10^-4^ (3.051×10^-4^–9.000×10^-4^) |
|  |  | Maximum likelihood | 1511.1 | 8.058×10^-4^ |
| Movement protein | 485 | Bayesian | 1786.5 (1647.0–1903.2) | 1.278×10^-3^ (9.075×10^-4^–1.658×10^-3^) |
|  |  | Bayesian (with discrete location states) | 1832.6 (1743.5–1911.3) | 8.788×10^-4^ (6.848×10^-4^–1.088×10^-3^) |
|  |  | Maximum likelihood | 1963.7 | 6.784×10^-4^ |
|  | 458 | Bayesian | 1807.3 (1687.7–1902.15) | 1.407×10^-3^ (1.040×10^-3^–1.786×10^-3^) |
|  |  | Bayesian (with discrete location states) | 1918.3 (1868.4–1959.5) | 1.609×10^-3^ (1.173×10^-3^–2.067×10^-3^) |
|  |  | Maximum likelihood | 1961.5 | 1.100×10^-3^ |
|  | 415 | Bayesian | 1742.2 (1576.7–1882.8) | 8.671×10^-4^ (6.143×10^-4^–1.130×10^-3^) |
|  |  | Bayesian (with discrete location states) | 1811.2 (1707.1–1902.8) | 6.796×10^-4^ (5.062×10^-4^–8.576×10^-4^) |
|  |  | Maximum likelihood | 1626.6 | 6.309×10^-4^ |
|  | 385 | Bayesian | 1748.1 (1580.2–1894.4) | 1.002×10^-3^ (6.996×10^-4^–1.344×10^-3^) |
|  |  | Bayesian (with discrete location states) | 1864.0 (1780.0–1930.3) | 1.185×10^-3^ (8.725×10^-4^–1.493×10^-3^) |
|  |  | Maximum likelihood | 1955.1 | 4.648×10^-3^ |
|  | 355 | Bayesian | 1685.9 (1484.8–1857.7) | 8.303×10^-4^ (5.365×10^-4^–1.151×10^-4^) |
|  |  | Bayesian (with discrete location states) | 1817.3 (1708.5–1907.6) | 9.876×10^-4^ (6.847×10^-4^–1.298×10^-3^) |
|  |  | Maximum likelihood | 1949.4 | 4.228×10^-3^ |
|  | 325 | Bayesian | 1524.6 (1095.3–1817.7) | 6.011×10^-4^ (2.692×10^-4^–9.062×10^-4^) |
|  |  | Bayesian (with discrete location states) | 1825.7 (1720.7–1910.1) | 9.984×10^-4^ (7.323×10^-4^–1.304×10^-3^) |
|  |  | Maximum likelihood | 1947.7 | 4.055×10^-3^ |

*^a^* MRCA, most recent common ancestor.

Supplementary Table. 4 Statistics of the regional origin of BYDV.

| Genes | Date sets | Root state posterior probabilities | | | | | | |
| --- | --- | --- | --- | --- | --- | --- | --- | --- |
|  |  | Brazil | China | Europe | New Zealand | Pakistan | South Korea | USA |
| Coat protein | 379 | 0.0142 | 0.0141 | 0.0732 | 0.1281 | 0.0203 | 0.0140 | 0.7122 |
|  | 356 | 0.0179 | 0.0279 | 0.1066 | 0.1709 | 0.0567 | 0.0161 | 0.6038 |
|  | 333 | 0.0217 | 0.0866 | 0.0609 | 0.1428 | 0.0298 | 0.0326 | 0.5919 |
|  | 308 | 0.0228 | 0.0244 | 0.1142 | 0.1545 | 0.0488 | 0.0203 | 0.5496 |
|  | 283 | 0.0283 | 0.0357 | 0.1279 | 0.2069 | 0.0439 | 0.0247 | 0.4954 |
|  | 258 | 0.0316 | 0.0524 | 0.1048 | 0.2136 | 0.0532 | 0.0295 | 0.4657 |
| Movement protein | 485 | 0.0067 | 0.2632 | 0.1805 | 0.0367 | 0.0351 | 0.0041 | 0.4737 |
|  | 458 | 0.0386 | 0.1545 | 0.1328 | 0.1345 | 0.0656 | 0.0097 | 0.4824 |
|  | 415 | 0.0101 | 0.0477 | 0.2235 | 0.0122 | 0.0948 | 0.0077 | 0.5346 |
|  | 385 | 0.0068 | 0.0308 | 0.1401 | 0.0752 | 0.0274 | 0.0103 | 0.7094 |
|  | 355 | 0.0136 | 0.0435 | 0.2724 | 0.0714 | 0.1423 | 0.0091 | 0.4358 |
|  | 325 | 0.0143 | 0.0348 | 0.1769 | 0.0904 | 0.1828 | 0.0089 | 0.4919 |

Supplementary Table. 5 Statistically supported migration rates of Barley yellow dwarf virus.

| Gene | From | To | Indicator *^a^* | Bayes factor *^b^* |
| --- | --- | --- | --- | --- |
| CP | Central China | East China | >1000 | 1.000 |
|  | Central China | Southwest China | >1000 | 0.999 |
|  | Germany | Estonia | >1000 | 0.999 |
|  | Middle Reaches of Yellow River | Central China | >1000 | 1.000 |
|  | Middle Reaches of Yellow River | East China | 24.492 | 0.704 |
|  | Middle Reaches of Yellow River | Northwest China | >1000 | 0.999 |
|  | Southwest China | Middle Reaches of Yellow River | 198.512 | 0.951 |
|  | Southwest China | Northwest China | 73.326 | 0.877 |
|  | The Unites States | Southwest China | 57.035 | 0.847 |
|  | The Unites States | Brazil | 11.388 | 0.525 |
|  | The Unites States | Germany | 32.974 | 0.762 |
|  | The Unites States | Pakistan | 377.172 | 0.973 |
|  | The Unites States | New Zealand | 111.397 | 0.915 |
| MP | Central China | East China | >1000 | 1.000 |
|  | Central China | Southwest China | >1000 | 0.999 |
|  | East China | South Korea | 46.159 | 0.818 |
|  | Germany | Estonia | >1000 | 0.998 |
|  | Germany | The Unites States | 56.621 | 0.846 |
|  | Middle Reaches of Yellow River | Central China | >1000 | 0.997 |
|  | Middle Reaches of Yellow River | East China | 67.268 | 0.867 |
|  | Middle Reaches of Yellow River | Northwest China | >1000 | 1.000 |
|  | New Zealand | East China | 61.469 | 0.857 |
|  | Northwest China | Estonia | 11.388 | 0.525 |
|  | Southwest China | Middle Reaches of Yellow River | >1000 | 0.998 |
|  | Southwest China | Germany | 31.823 | 0.756 |
|  | Southwest China | Northwest China | 35.295 | 0.774 |
|  | Southwest China | South Korea | 12.624 | 0.551 |
|  | The Unites States | Brazil | 9.588 | 0.513 |
|  | The Unites States | Southwest China | >1000 | 0.990 |
|  | The Unites States | Pakistan | 122.953 | 0.923 |
|  | The Unites States | New Zealand | >1000 | 0.998 |

*^a^* Decisive rates: BF>1,000; Very strongly supported rates: 100≤BF<1,000; Strongly supported rates: 10≤BF<100; Supported rates: 5≤BF<10.

*^b^* Statistically supported migration rates with a mean indicator of >0.5:
